# Functional and genomic adaptations of blood monocytes to pregravid obesity during pregnancy

**DOI:** 10.1016/j.isci.2021.102690

**Published:** 2021-06-04

**Authors:** Suhas Sureshchandra, Nicole E. Marshall, Norma Mendoza, Allen Jankeel, Michael Z. Zulu, Ilhem Messaoudi

**Affiliations:** 1 Department of Molecular Biology and Biochemistry, University of California, 2400 Biological Sciences III, Irvine, CA 92697, USA; 2 Institute for Immunology, University of California, Irvine, CA 92697, USA; 3 Center for Virus Research, University of California, Irvine, CA 92697, USA; 4 Maternal-Fetal Medicine, Oregon Health and Science University, Portland, OR 97239, USA

**Keywords:** Immunology, Pregnancy, Immune system

## Abstract

Pregravid obesity is associated with several adverse maternal health outcomes, such as increased risk of infection, suggesting an altered immunological state. However, the mechanisms by which obesity disrupts the pregnancy “immune clock” are still unknown. Here, we profiled circulating immune mediators, immune cell subset frequencies, and peripheral immune responses during the first and third trimesters of pregnancy in lean and obese mothers. While both Th1 and Th2 cytokines were elevated with pregnancy regardless of BMI, obese subjects had dysregulated myeloid factors in circulation at term. Pregnancy in lean subjects was associated with enhanced monocyte activation, augmented chromatin accessibility at inflammatory loci, and heightened responses to LPS. Pregravid obesity disrupted this trajectory, resulting in a lack of transcriptional, epigenetic, and metabolic changes strongly suggesting a skewing toward innate immune tolerance. These findings provide novel insight into the increased susceptibility to infections in women with obesity during pregnancy and following cesarean delivery.

## Highlights

• Pregnancy is associated with activation and enhanced responses of monocytes
• Heightened monocyte response is associated with epigenetic adaptions
• Pregravid obesity leads to a state akin to LPS tolerance in monocytes
• Pregravid obesity is associated with a lack of epigenetic and metabolic plasticity

## Graphical abstract

**Figure:**
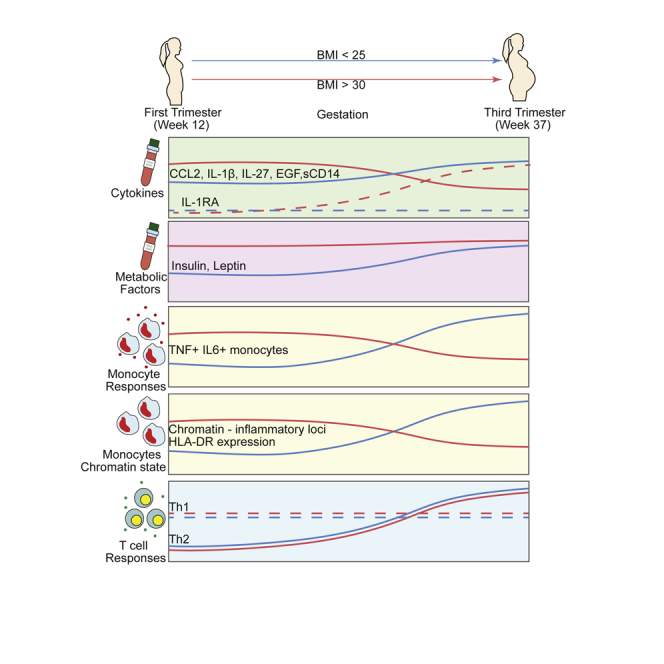

## Introduction

The maternal immune system undergoes several fine-tuned adaptations throughout pregnancy dubbed the “immunological clock of pregnancy” (Mor and Cardenas, 2010). These adaptations are believed to facilitate implantation, fetal tolerance, fetal growth and development, and finally labor and parturition without compromising protection against microbial infections (Mor et al., 2011). These changes do not necessarily follow waves of pro- and anti-inflammatory phases (Mor and Cardenas, 2010), but rather a progressive activation of signaling molecules (Aghaeepour et al., 2017) and alterations in the circulating proteome (Aghaeepour et al., 2018). Specifically, over the course of a healthy pregnancy, the peripheral adaptive immune system is skewed toward Th2/Treg responses and away from Th1/Th17 responses (Luppi et al., 2002b; Polese et al., 2014; Sacks et al., 2003), while innate immune cells are progressively activated (Aghaeepour et al., 2017). This activation is manifested through higher production of pro-inflammatory cytokines IL-6, IL-1β, and IL-12 by peripheral blood mononuclear cells (PBMC) following *ex vivo* stimulation with LPS and bacteria (Aghaeepour et al., 2017; Faas et al., 2014; Luppi et al., 2002a; Naccasha et al., 2001; Sacks et al., 1998, 2003) or viral particles (Kay et al., 2014; Le Gars et al., 2016). However, the precise mechanisms underlying this progressive activation of the innate immune branch remain poorly defined.

A third of women of reproductive age in the US meet the definition of obese (body mass index (BMI)>30) (Flegal et al., 2016), making pregravid obesity one of the most critical and common health challenges during pregnancy. High pregravid BMI increases the risk for gestational diabetes (Chu et al., 2007; Torloni et al., 2009), gestational hypertension, preeclampsia (Huda et al., 2014; Wang et al., 2013), early pregnancy loss, placental abruption, abnormal fetal growth, premature labor, and stillbirth (Aune et al., 2014; Kim et al., 2016). High pregravid BMI is also an independent risk for infection during pregnancy (urinary and genital tract infections, sepsis) (Sebire et al., 2001), labor (chorioamnionitis) (Cnattingius et al., 2013; Hadley et al., 2019), and post-partum (surgical site infections following cesarean) (Conner et al., 2014; McLean et al., 2012).

These adverse outcomes suggest a dysregulated immune status in pregnant women with obesity, but the impact of pregravid obesity on the pregnancy “immune clock” remains poorly defined. Pregravid obesity is marked by elevated circulating levels of inflammatory mediators C-reactive protein (CRP) and IL-6 (Basu et al., 2011a; Sureshchandra et al., 2018), subclinical endotoxemia, and signs of heightened inflammation in the adipose tissue compartment (Basu et al., 2011a). Increased exposure to subclinical levels of LPS with obesity could potentially alter both the phenotype and fitness of innate immune cells. Indeed, we recently reported alterations in the secretion of immune mediators, especially those expressed by monocytes and dendritic cells (DCs), following *ex vivo* stimulation with TLR agonists of PBMC collected at gestational week 37 from subjects with obesity (Sureshchandra et al., 2018).

Amongst innate immune cells, monocytes and macrophages play critical roles at various stages of pregnancy. Circulating monocytes play seminal roles in implantation, placentation, and labor, while decidual monocyte-derived macrophages play crucial roles in angiogenesis, tissue development, and wound healing at the maternal-fetal interface (Basu et al., 2011b; Mor and Abrahams, 2003; Mor et al., 2011). Monocytes/macrophages have also been implicated in several obesity-associated complications during pregnancy such as preeclampsia (Faas et al., 2014), preterm labor (Gomez-Lopez et al., 2014), poor placental development (Faas and de Vos, 2017), and chorioamnionitis (Ben Amara et al., 2013). However, several questions remain unaddressed: (1) How does pregravid obesity alter monocyte phenotype and function throughout pregnancy; (2) what are the cellular and molecular mechanisms underlying those changes?

Therefore, in this study, we defined longitudinal changes in circulating immune mediators, peripheral innate immune cell subsets, and responses of monocytes and DCs to *ex vivo* stimulation during early and late stages of gestation in both lean and obese women to elucidate the impact of pregravid obesity on the “pregnancy immunological clock” using a combination of immunological, transcriptional, and epigenetic analyses. We report a gestation-associated trajectory of monocyte activation that is accompanied by an enhanced response to LPS and poising of the epigenome toward a state of heightened activation. Pregravid obesity disrupts this trajectory of maternal monocyte activation, skewing them toward an immunoregulatory phenotype characterized by attenuated responses to LPS and lack of epigenetic and metabolic plasticity observed in lean pregnancy. This dysregulation in monocyte activation with pregravid obesity was accompanied by systemic changes in circulating cytokines and chemokines consistent with a regulatory environment. Collectively, these findings provide novel insight into mechanisms by which pregravid obesity disrupt pregnancy-associated adaptations that mediate tolerance while protecting the developing fetus and pregnant women against microbial challenges.

## Results

### Pregnancy and pregravid obesity alter the circulating metabolic and inflammatory environment

**Figure 1:**
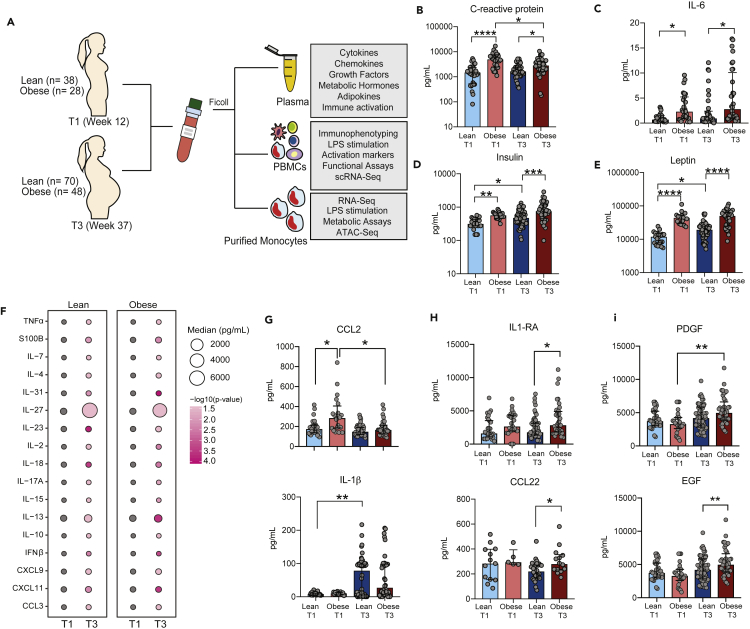
Experimental design and longitudinal measurements of circulating maternal immune and metabolic hormones (A) Blood samples were obtained from pregnant women during early (week 12 – T1) and late (week 37 – T3) pregnancy to assess the impact of pregravid obesity on maternal immunity. (B and C) Bar graphs depicting changes in plasma levels of inflammatory mediators (B) CRP and (C) IL-6 measured using high sensitivity ELISA (Lean T1 n = 32, Obese T1 n = 28, Lean T3 n = 42, Obese T3 n = 40). (D and E) Circulating levels of metabolic hormones (D) insulin, (E) leptin were measured using Luminex (Lean T1 n = 20, Obese T1 n = 18, Lean T3 n = 57, Obese T3 n = 38). Levels of significance: ∗ - p<0.05, ∗∗ - p<0.01, ∗∗∗p<0.001, ∗∗∗∗ - p< 0.0001. Bars denote median with interquartile ranges. (F) Bubble plot representing changes in immune mediator levels in plasma with gestation (Lean T1 n = 20, Obese T1 n = 18, Lean T3 n = 29, Obese T3 n = 26). The size of the bubble represents median values of analytes in pg/mL (log2 transformed). The colors at T3 represent the levels of significance relative to T1. (G and H) Bar graphs comparing plasma levels of (H) pro-inflammatory myeloid factors CCL2 and IL-1β and (H) regulatory cytokine (IL-1RA), chemokine (CCL22), and growth factors (PDGF and EGF) (Lean T1 n = 20, Obese T1 n = 18, Lean T3 n = 29, Obese T3 n = 26). Levels of significance: ∗- p<0.05, ∗∗ - p<0.01; ∗∗∗ - p<0.001; ∗∗∗∗- p<0.0001.

Both pre-pregnancy BMI and fat mass were recorded to stratify subjects as lean (BMI <25) and obese (BMI >30). Patient demographics are summarized in Table 1. Pregravid BMI correlated strongly with fat mass percentages, both at T1 and T3 (Figure S1A). Gestational weight gain was significantly lower in the obese group (Figure S1B). Since obesity is characterized by changes in metabolic profile and chronic low-grade inflammation, we first measured plasma levels of IL-6, CRP, and metabolic hormones (Figure 1A). At both T1 and T3, high pregravid BMI was associated with elevated circulating CRP (Figure 1B) (3-fold at T1, 1.5-fold at T3) and IL-6 (2.9-fold at T1, 2.7-fold at T3) (Figure 1C) levels. Significantly elevated systemic levels of insulin (2-fold) (Figure 1D) and leptin (4-fold) (Figure 1E) at T1 and T3 were observed in the obese group. Interestingly, insulin and leptin levels remained unchanged in the obese group but increased with gestation in the lean group (Figures 1D and 1E). Plasma levels of resistin were also higher in the obese group at both time points (4-fold and T1 vs. 1.5-fold at T2) but did not increase with pregnancy in either group (Figure S1C). Finally, plasma levels of the adipokines adipsin (Figure S1D) and adiponectin (Figure S1E) increased with pregnancy in both groups (1.5-fold for adipsin and 2-fold for adiponectin), but no group differences were noted. No differences were detected in plasma levels of gut hormone peptide YY (PYY) with either pregnancy or pregravid obesity (Figure S1F). Collectively, these observations suggest that metabolic adaptations are a crucial part of a healthy pregnancy that likely support fetal growth and development and that pregravid obesity during pregnancy disrupts these adaptations and results in chronic low-grade inflammation.

**Table 1:** Cohort details – maternal and fetal characteristics

Parameter	Lean	Obese	p value
Sample size (n)	69	48	
Race (n)			ns
American Indian/Alaskan native:	2	4	
Asian American:	6	1	
African American:	0	6	
Pacific Islander:	2	0	
White/Caucasian:	64	37	
Hispanic:	6	8	
BMI (kg/m^2^)	21.9 ± 1.7	35.2 ± 5.2	<0.0001
Fat mass at 12 weeks (kg)	17.3 ± 4.1	42.6 ± 11.4	<0.0001
Fat mass at 37 weeks (kg)	22 ± 5.5	44.2 ± 11.1	<0.0001
Gestational weight gain (GWG)(kg)	15 ± 4.5	10.4 ± 7.6	<0.0001
Nulliparous (%)	58.2	66.7	ns
Gestational age at delivery (weeks)	39.4	39.7	ns
Fetal sex (male %)	47.2	48.4	ns
Fetal birthweight (kg)	3.43	3.52	ns
Mode of delivery (% vaginal)	75.7	73.1	ns

To further assess the impact of both pregnancy and obesity on circulating immune mediators, we profiled plasma cytokine, chemokine, and growth factor levels using a multiplex panel of myeloid and lymphoid factors. Independent of maternal BMI, pregnancy was associated with shifts in the circulating inflammatory environment from T1 to T3 (Figure 1F). Levels of both pro-inflammatory (TNFα, IL-2, IFNβ, IL-7, IL-17A, IL-18, IL-23) and regulatory (IL-4, IL-10, and IL-13) cytokines, as well as chemokines (CCL3, CXCL9, and CXCL11) significantly increased during pregnancy in both lean and obese subjects (Figure 1F). At T1, obesity was associated with a 2-fold increase of myeloid cell chemoattractants CCL2 (Figure 1G) and 1.5-fold increase in circulating CCL4. Levels of CCL2 decreased dramatically (50%) between T1 and T2 in the obese group but remained unchanged in the lean group (Figure 1G). In contrast, levels of CCL4, as well as the pro-inflammatory myeloid cell activator IL-27, increased with gestation in both groups, albeit more prominently in the lean group (5-fold in lean group vs. 3-fold in the obese group) (Figures S1G and S1H). Levels of the myeloid pro-inflammatory factor IL-1β only increased in the lean group (7-fold) with pregnancy (Figure 1F). Additionally, levels of regulatory factors IL-1RA, chemokine CCL22, and growth factors EGF were modestly increased in the obese group relative to the lean group at T3 alone (20-50%), while levels of PDGF were increased with pregnancy in the obese group (Figures 1H and 1I). Finally, a small (35%) but significant drop in levels of regulatory factor VEGF was observed in the lean group alone (Figure S1I) over the course of pregnancy.

We next correlated pregnancy-associated changes in circulating immune mediators with maternal BMI (Figure S1J) to identify key factors whose temporal changes were maternal BMI dependent. Our analyses revealed a positive association between maternal BMI and changes in pro-inflammatory factors leptin and IL-18 as well as regulatory factors IL-10 and PDGF (Figure S1J). Surprisingly, although CRP levels were elevated with obesity, they decreased between T1 and T3 (Figure 1B). This decrease negatively correlated with maternal BMI (Figure S1J).

Collectively, these observations suggest that healthy pregnancy is associated with a progressive enhancement of myeloid cell activation while pregravid obesity skews circulating myeloid cells toward a regulatory phenotype at term. This adaptation suggests potential mechanisms of limiting obesity-induced inflammation in pregnant women.

### Pregravid obesity alters the frequencies of monocyte subsets and disrupts pregnancy-associated activation trajectory of circulating monocytes

Amongst the factors that exhibited statistically significant differences between the lean and obese groups with obesity at T3 were myeloid factors, including CCL4, IL-1β, IL-1RA, and IL-27. We, therefore, investigated the impact of pregnancy and obesity on the frequency and function of circulating immune cells. Complete blood cell (CBC) counts revealed a 25% increase in frequencies of white blood cells (WBC) with pregnancy and with pregravid obesity (Figure S2A). These differences were primarily driven by granulocytes, with no significant differences in lymphocyte or monocyte numbers (Figure S2A).

**Figure 2:**
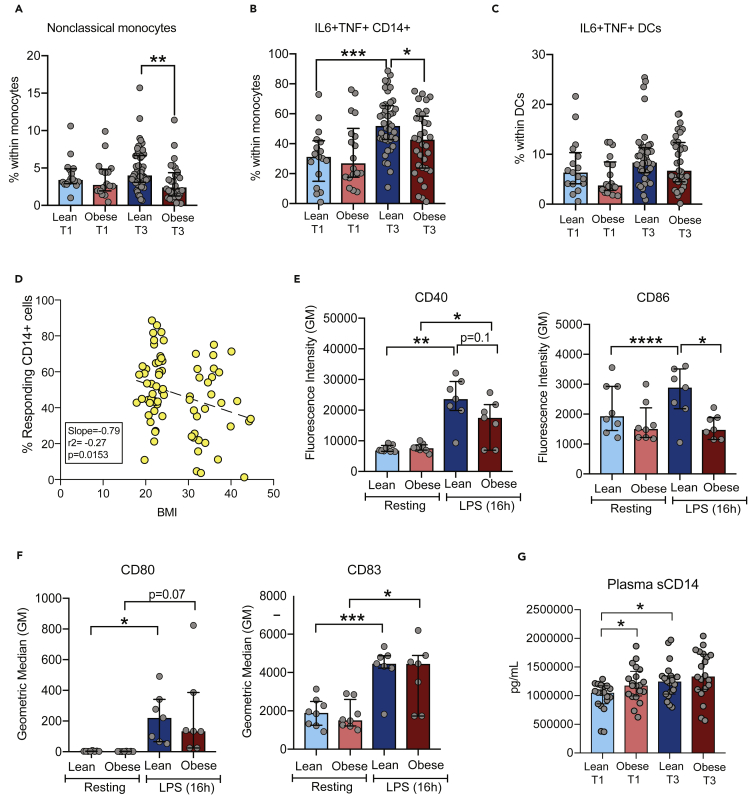
Pregnancy and obesity-associated changes in innate immune phenotype and *ex vivo* response (A) Bar graph depicting percentages of nonclassical subset (CD14^+^CD16^++^) within monocytes (Lean T1 n = 18, Obese T1 n = 18, Lean T3 n = 50, Obese T3 n = 30). (B and C) Percentages of IL6 and TNF producing (B) monocytes and (C) dendritic cells (DCs) following 16hr LPS stimulation (Lean T1 n = 17, Obese T1 n = 17, Lean T3 n = 43, Obese T3 n = 33). (D) Linear regression analysis of maternal BMI and monocyte responses to LPS at T3. Deviations in the slope of regression line were tested using F-test. (E and F) Surface expression of activation markers (E) CD40, CD86, and (F) CD80, and CD83 on monocytes following 16hr LPS stimulation at T3 time point (Lean T3 n = 8, Obese T3 n = 8). (G) Plasma levels of soluble CD14 measured at both time points using ELISA (n = 19/group). Levels of significance: ∗ - p<0.05, ∗∗ - p<0.01. Bars denote median and interquartile ranges.

We next used multi-color flow cytometry to measure frequencies of major leukocytes and their subsets within PBMC (Figure S2B). No changes in major cell populations (Figures S2C–S2E) were observed either with pregnancy or pregravid obesity except for a decrease in dendritic cells (DC) frequency in the lean group between T1 and T3 (Figure S2D). In mothers with obesity, pregnancy was associated with a redistribution of DC subsets, with higher mDC (Figure S2F) but lower pDC subsets at T3 (Figure S2G). Pregravid obesity was also associated with a 25% reduction in the proportion of nonclassical (CD16++) subset of monocytes (Figure 2A), in line with the attenuation in circulating mediators typically associated with activation of myeloid cells, especially monocytes.

Although the frequency of NK cells was unchanged with pregnancy or pregravid obesity (Figure S2E), the relative abundance of CD56^bright^CD16^+^ NK cells increased by 70% with pregnancy in both groups (Figure S2H). Very few changes in the frequency of lymphocytes were observed with pregnancy or obesity (Figure S3). Pregravid obesity was associated with no changes in the frequency of B cells (Figure S3A), but with a 30% drop in the frequency of memory and marginal-zone (MZ) like B cells (Figure S3D) with pregnancy. Pregnancy in the lean group was also accompanied by a 10% decrease in B cell subsets (naive (p = 0.07); MZ like B cells (p<0.0001) (Figure S3D) and a 50% drop in frequencies of transitional effector memory CD4+ T cells (Figure S3E). Changes within these specific T and B cell subsets without major changes in overall lymphocyte frequencies suggest a redistribution of maternal lymphocytes over the course of pregnancy.

Given the systemic changes in immune mediators, we asked if pregnancy or obesity alter the state of immune activation. We began by testing the ability of innate immune cells and T cells to respond to *ex vivo* stimulation with lipopolysaccharide (LPS) and anti-CD3/CD28, respectively (Figure 1A) using intracellular staining (Figure S4A). Pregnancy in lean women was associated with increased frequency of IL6^+^TNF^+^ producing monocytes (70% increase) (Figure 2B), but not dendritic cells (Figure 2C), in response to LPS stimulation. This enhanced monocyte response was absent in the obese group resulting in fewer responding monocytes compared to the lean group at T3 (Figure 2C). This difference in LPS response was not mediated by alterations in cell death between the two groups. Interestingly, the frequency of responding monocytes at T3 negatively correlated with maternal pregravid BMI (r^2^ =-0.27, p = 0.015) (Figure 2D). Enhanced response to LPS at T3 by monocytes from the lean group was accompanied by the induction of activation markers CD40 (3-fold) and CD86 (20%) (Figure 2E) and a 20% increase in plasma levels of soluble CD14 (sCD14), a surrogate for *in vivo* monocyte activation (Figure 2G). Activation markers CD80 (60-fold) and CD83 (2-fold) were up-regulated to similar levels in both groups (Figure 2F). Cytokine production by T cells remained essentially unchanged with pregnancy and pregravid obesity (Figures S4A and S4B) except for enhanced Th2 responses (IL-4+ CD4 T cells) (Figure S4B).

### Single cell RNA-seq reveals significant shifts in monocyte phenotypes at T3

To better understand the impact of pregravid obesity on monocyte activation with pregnancy, we first carried out bulk RNA-Seq analysis on purified monocytes obtained from T1 and T3 from lean and obese subjects (n = 3/time point). Pairwise longitudinal comparison of the transcriptome of resting monocytes from the lean group showed significant changes, with increased expression of MHC molecules (*B2M*, *HLA-DRA*) and proinflammatory genes (*VCAN*, *LYZ*) (p value <0.001) at T3 relative to T1 (Figure S5A). On the other hand, monocytes from mothers with obesity (n = 3/time point) exhibited suppression of genes involved in interferon signaling (*STAT1*, *GBP5*, *PSMB8*) and response to reactive oxygen species (*STRBP*, *HEBP2*, *TRAP1, TRPA1*) (p value <0.001) with gestational age (Figure S5B).

**Figure 3:**
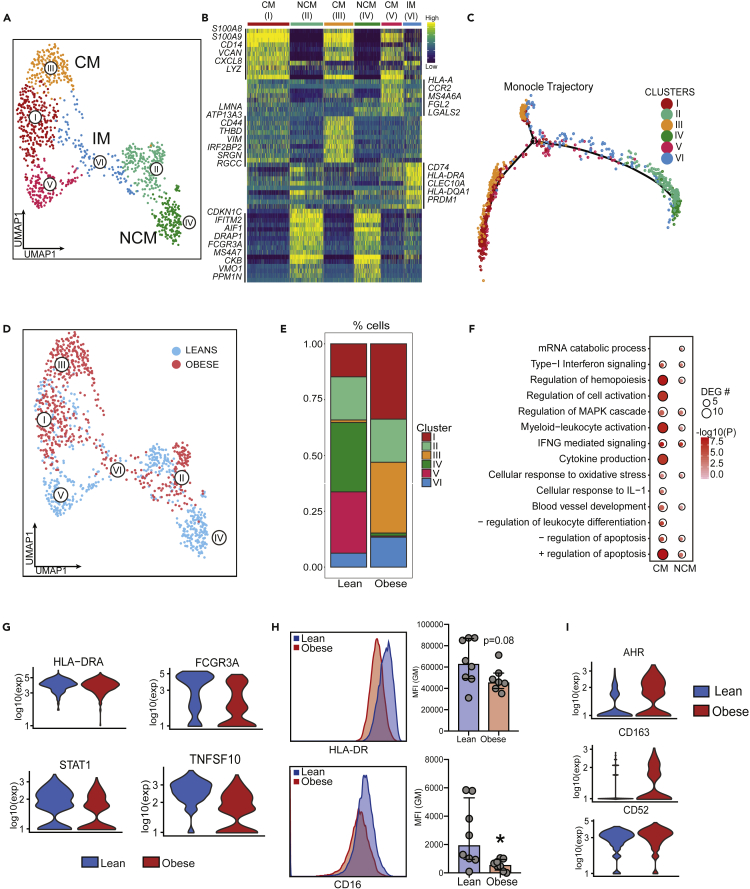
Single-cell profiles of blood monocytes at delivery (A) UMAP of monocytes following extraction of CD14^high^ clusters from UMAP of PBMC from lean (n = 2) and obese (n = 2) subjects, colored by cell state (clusters), and annotated by cluster numbers and monocyte subsets (CM-classical monocytes, IM – intermediate, NCM – nonclassical monocytes). (B) Heatmap showing expression patterns of the 10 most distinguishing markers per cell state (yellow = high expression; green = intermediate expression; blue = low expression) phenotype. Select markers are indicated in the margins. (C) Trajectory analysis of monocyte with cells colored by their original cluster designation. (D) UMAP visualization of monocytes, colored by the group. (E) Relative frequencies of cells within each UMAP cluster in either group. (F) Functional enrichment of genes differentially expressed with pregravid obesity in classical and nonclassical monocyte subsets. Bubble size and color are indicative of the number of genes within each ontology term and the significance of its enrichment. (G) Violin plots showing log-transformed, normalized expression levels for select genes down-regulated with pregravid obesity. (H) Surface expression of HLA-DR and CD16 using flow cytometry. Error bars represent medians with interquartile range. (I) Violin plots showing log-transformed, normalized expression levels for select genes up-regulated with pregravid obesity.

Given the few transcriptional changes detected using bulk RNA-Seq and the significant differences in monocyte responses to LPS stimulation observed at T3, we performed single-cell RNA-sequencing of total PBMC (n = 2/group) obtained at delivery (T3). After concatenating all 4 libraries, we computationally extracted the monocyte clusters (∼600 cells/group – total 2400 cells) based on the expression of *CD14* (clusters 7, 11, and 16 in the initial UMAP of PBMC; Figure S5C; Table S1). Following subsequent iterations of doublet removal and UMAP clustering, we identified 6 major clusters of monocytes (Figure 3A) representing the three subsets traditionally identified by flow cytometry based on the expression of *CD14*, *FCGRA* (CD16), and *HLA-DRA* (Figure S5D): Classical monocytes (CM – clusters I, III, V); nonclassical monocytes (NCM - clusters II and IV); and intermediate (IM-cluster VI) (Figure 3B). Trajectory analysis using Monocle confirmed this classification, placing nonclassical monocytes at the end of the pseudotime with intermediate monocytes transitioning between classical and nonclassical subsets (Figures 3C and S5E). Pregravid obesity was associated with a dramatic drop in the number of nonclassical monocytes (especially cluster IV, 27% in leans vs. 7% in obese) (Figures 3D and 3E) in line with our flow cytometry data (Figure 2A). We also detected a significant increase in classical monocytes mediated by a substantial increase in cluster III but a decrease in cluster V (Figures 3D and 3E). Expansion of classical monocytes (cluster III, 3% in leans vs. 30% in obese) expressing markers of insulin resistance (*CD44*, *LMNA*, *THBD*) (Figure 3B), suggests metabolic adaptations to maternal obesity at T3, whereas reduction of classical monocytes (cluster V, 20% in leans vs. 1% in obese) (Figures 3B, 3D and 3E) expressing pro-inflammatory genes (*VCAN*, S100A9, *LYZ*) (Figure 3B) supports the inadequate *ex vivo* responses to LPS observed in the obese group at term.

Differential gene expression analysis within both classical and nonclassical clusters revealed transcriptional changes associated with immune activation, signaling, differentiation, and apoptosis with obesity (Figure 3F, Table S1). Specifically, we detected down-regulation of *HLA-DRA* (1.38-fold), *STAT1* (1.3-fold), *TNFSF10* (1.7-fold), and *FCGR3A* (fold reduction 1.65) (Figure 3G) with pregravid obesity. Reduced surface expression HLA-DR (0.5-fold) and CD16 (5-fold) (encoded by *FCGR3A*) with pregravid obesity were confirmed using flow cytometry (Figure 3H). As observed with bulk RNA-sequencing (Figure S5B), we observed a global suppression of interferon signaling pathways with pregravid obesity across all clusters (Figure S5F) as exemplified by reduced expression of interferon-stimulated genes (*IFIT3*, *ISG15*, *OAS1*) (Figure S5G). On the other hand, pregravid obesity was associated with up-regulation of immunoregulatory molecules such as *AHR* (1.44-fold), *CD52* (1.21-fold), and *CD163* (1.8-fold) (Figure 3I). These transcriptional differences at single cell resolution further support the conclusion that pregravid obesity results in transcriptional skewing of circulating monocytes toward a regulatory phenotype.

### Defects in monocyte responses with maternal obesity at T3 are cell intrinsic

**Figure 4:**
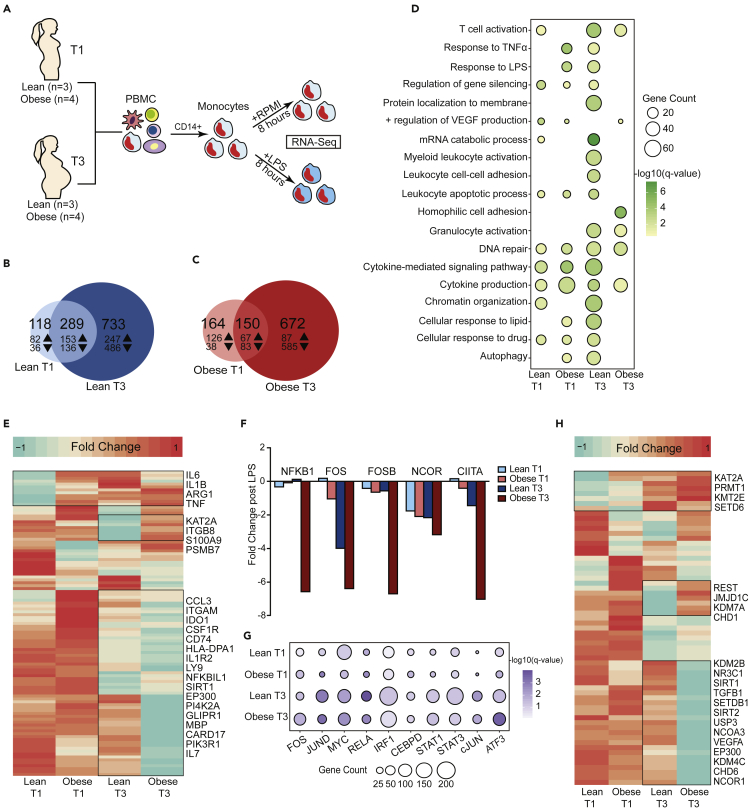
Cell intrinsic defects in monocyte responses to LPS with maternal obesity (A) Experimental design for RNA-seq. (B and C) Venn diagrams comparing LPS-induced differentially expressed genes (DEG) between T1 and T3 in purified monocytes from (B) lean and (C) obese (n = 4/group and time point) subjects following 8hr LPS stimulation. Numbers of up- and down-regulated DEG (FDR≤0.05) are annotated with corresponding arrows. (D) Bubble plot representing gene ontology (GO) terms enriched following LPS stimulation in all four groups following LPS stimulation. Both up- and down-regulated genes are included. The size of the bubble represents numbers of genes mapping to the term, while color indicates the level of significance. (E) Heatmap comparing fold changes of DEG (both up- and down-regulated) that mapped to GO terms “cytokine production” and “myeloid leukocyte activation”. Green indicates down-regulation while orange indicates up-regulation. (F) Fold change of key nuclear factors significantly down-regulated with LPS. (G) Bubble plots representing the number of DEG regulated by specific LPS inducible transcription factors predicted by ChEA3. The size of the bubble represents the numbers of DEG regulated by each transcription factor. The color of the bubble represents the level of significance for each prediction. (H) Heatmap comparing fold changes of DEG (both up- and down-regulated) that mapped to GO terms “Chromatin organization”. Green indicates down-regulation while orange indicates up-regulation.

We next investigated whether the reduction in cytokine production by circulating monocytes in response to LPS with pregravid obesity is cell-intrinsic. Monocytes were isolated from PBMC (Figure S6A) obtained from lean subjects and those with obesity at T1 and T3 and stimulated with LPS for 8 hr (Figure 4A). Direct comparison of LPS responsive DEG at T1 (Figure S6B) and T3 (Figure S6C) revealed a subset of genes that were uniquely down-regulated at T3 in the obese group (Figure S6C, Table S2). Although gene expression responses to LPS were more robust at the T3 time point compared to T1 for both the lean (Figure 4B) and obese groups (Figure 4C), DEG detected at T3 in the obese group were predominantly down-regulated (Figures 4C and S6C). Moreover, functional enrichment revealed a higher proportion of DEG detected at T3 in the lean group enriched to GO terms associated with myeloid activation, leukocyte adhesion, and cytokine signaling (Figure 4D).

In line with these observations, we detected lower induction of key inflammatory genes *IL6*, *IL1B*, *TNF*, and *CCL3* (Figure 4E) in the obese group. Furthermore, at T3, we observed reduced expression of key transcription factors *NFKB1*, *FOSB*, *NCOR*, *CIITA* (Figure 4F) with pregravid obesity (2-4 fold drop in leans vs. 4-6 fold drop in obese). These findings were supported by transcription factor analysis using ChEA3, which revealed a reduced number of RELA, IRF, and STAT-regulated DEG with pregravid obesity, particularly at T3 (Figure 4G). On the other hand, the number of LPS-responsive genes regulated by TF IRF1, STAT1, and STAT3 was increased in the lean group at T3 compared to T1 (Figure 4G). Finally, we report poor induction of key regulators of chromatin reorganization (*KDM2B*, *SIRT1*, *SIRT2*, *EP300*, *KDM4C*) following LPS stimulation in the obese group at T3 (Figures 4D and 4H), suggestive of epigenetic constraints to immune activation.

### Pregravid obesity disrupts pregnancy-associated epigenetic trajectory of circulating monocytes

**Figure 5:**
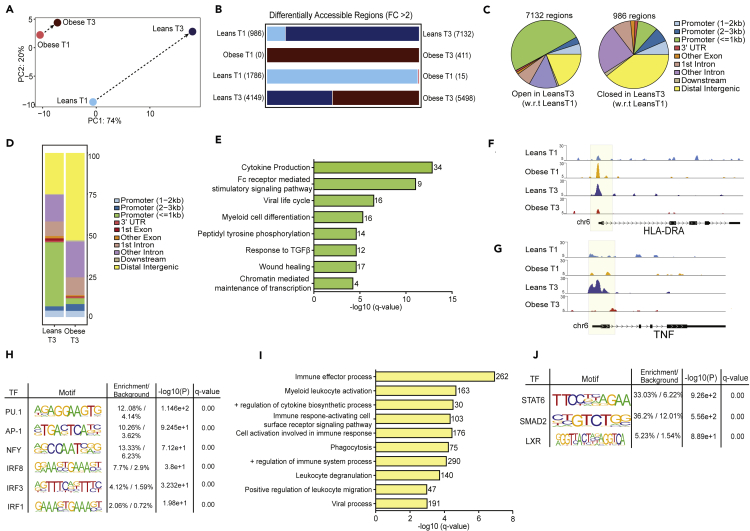
Epigenetic adaptations with pregnancy and maternal obesity (A) PCA of chromatin accessibility profiles in monocytes isolated at T1 and T3 from lean and obese subjects (n = 4/group and time point). Arrows represent trajectories from T1 to T3. (B) Bar graphs with numbers of differentially accessible regions (DAR) identified in each comparison. Numbers in parentheses denote open regions identified in a particular group relative to the comparison group. (C) Pie charts showing genomic contexts of loci identified as DAR with gestation in the lean group. (D) Genomic annotations of 4149 and 5,498 DAR identified as open in lean and obese groups, respectively, at T3. (E) Functional enrichment of genes regulated by promoter-associated DAR open in lean group relative to obese group (FC>2). Genes associated with these regions are quantified next to each term. (F and G) WashU Epigenome tracks for (F) *HLA-DRA* and (G) *TNF* locus with promoter vicinity highlighted in yellow. (H) Enrichment of motifs in promoter associated open DAR in the lean group relative to the obese group at T3. Only motifs identified in myeloid cells are included in the table. (I) Gene ontologies of intergenic associations identified by GREAT as significantly open in the lean group relative to the obese group at T3. (J) Enrichment of motifs in open intergenic DAR in the obese group relative to the lean group at T3. Only motifs identified in myeloid cells are included in the table.

We next asked if there exists a pregnancy-associated epigenetic clock that explains the enhanced monocyte responses to LPS at T3 relative to T1 in lean subjects. We therefore profiled open chromatin of purified resting monocytes isolated from lean and obese groups at both time points using ATAC-Seq (Figure 1A). Principal component analysis revealed a significant shift in ATAC profiles of monocytes from T1 to T3 in lean subjects (Figure 5A, Table S3), with 7132 regions open at T3 relative to T1 (Figure 5B), most of which overlap the promoter regions (Figure 5C). Promoter regions open in monocytes from lean subjects at T3 relative to T1 regulated genes involved in leukocyte activation, myeloid differentiation, and regulation of oxidative stress (Figure S7A). Furthermore, intergenic regions open in monocytes from lean subjects at T3 relative to T1 regulated genes involved in mounting an inflammatory response to lipids and LPS (Figure S7B), suggesting epigenetic adaptations in response to metabolic changes during a healthy pregnancy.

In contrast, limited changes in chromatin accessibility were noted between T1 and T3 in monocytes from subjects with obesity (Figures 5A and 5B, Table S4). Direct comparison of chromatin accessibility patterns in monocytes from lean and obese subjects revealed more dramatic differences at T3 (Figure 5D) than at T1 (Figure S7C). Specifically, at T3, 40% of loci open in the lean group relative to the obese group overlapped promoter regions of genes involved in cytokine production, Fc receptor-mediated signaling and wound healing (Figure 5E). This is further illustrated by pileups of the loci of inflammatory genes *HLA-DRA* (Figure 5F), *TNF* (Figure 5G), *IL6ST* (Figure S7D), and activation marker *CD80* (Figure S7E). Finally, motif analysis revealed that promoter regions open in monocytes from lean subjects at T3 (closed in monocytes from subjects with obesity) harbor binding sites for transcription factors PU.1 and AP-1 and interferon regulators IRF1, IRF3, and IRF8. The limited number of promoter-associated regions that were open in monocytes from obese subjects relative to those from lean subjects at T3 (Figure 5D) regulated genes primarily involved in histone methylation and metabolic processes (Figures S7F and S7G).

GREAT enrichment of intergenic loci closed in the obese group at T3 relative to leans (Figure 5H) revealed several associations with immune effector process, phagocytosis, cytokine responses, and immune activation (Figure 5I). Functional enrichment using GREAT of the intergenic regions open in monocytes from obese subjects relative to lean subjects at T3 (Figure 5D) did not return any significant GO processes. On the other hand, motif analysis suggests an increased abundance of binding sites for TF that govern the development of regulatory M2-like macrophages, notably STAT2 (33% enrichment over 6% background) and LXR (5% enrichment over 1.5% background) (Figure 5J). These observations indicate that pregravid obesity disrupts the “pregnancy epigenetic clock” that results in monocyte activation during a healthy lean pregnancy. Rather, epigenetic changes within monocytes from pregnant subjects with obesity suggest functional skewing of circulating monocytes toward M2-like regulatory cells.

### Pregravid obesity is associated with metabolic and functional rewiring of circulating monocytes at term

**Figure 6:**
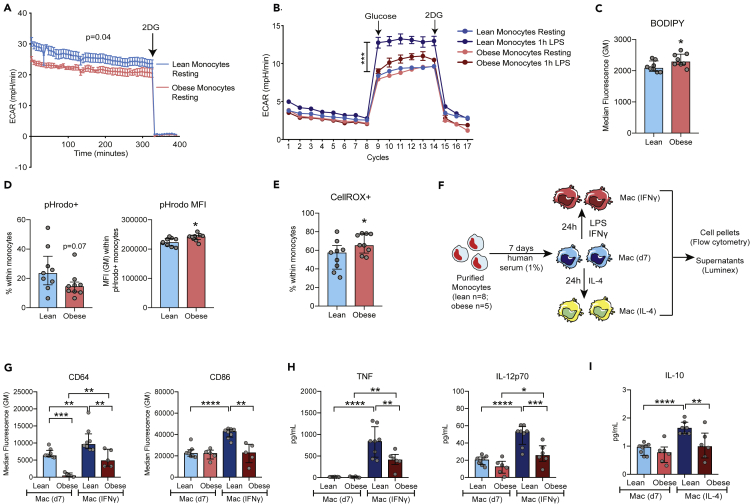
Metabolic and functional reprogramming of monocytes with maternal obesity at term (A) Mean ECAR of purified monocytes from lean (blue) and obese (red) group (n = 3/group) under basal conditions using glycolytic rate assay. (B) Mean ECAR of LPS activated monocytes (acute injection) from lean (blue) and obese (red) under glucose-free conditions, post glucose injection, and following injection of 2-Deoxy-D-glucose (2-DG; competitive inhibitor of glucose) using glycolytic stress assay. (C) Bar graphs comparing MFIs of BODIPY within CD14+ gate in PBMC (n = 8/group). (D) Bar graphs comparing pHrodo+ CD14+ cells following incubation of PBMC with pHrodo conjugated *E. coli* for 4 hr (left) and pHrodo signal within the pHrodo+ monocytes (right) (n = 9/group). (E) Cytosolic ROS readouts in LPS activated monocytes using FACS analysis of CellROX treated PBMC (n = 9/group). (F) Experimental design for monocyte differentiation and polarization assay (leans n = 8; obese n = 5). (G) Bar graphs comparing induction of surface CD64 and CD86 expression following LPS and IFNγ stimulation of monocyte-derived macrophages on day 7 post differentiation. (H) Secreted TNFα and IL-12p70 levels following LPS and IFNγ stimulation on day 7 using Luminex. (I) Secreted IL-10 levels following IL-4 stimulation on day 7 post differentiation.

We next asked if maternal obesity alters the metabolic/functional state of circulating monocytes at term. The initiation of proinflammatory effector mechanisms in monocytes requires a shift of cellular metabolism toward aerobic glycolysis. Therefore, we tested if the reduced response to LPS is associated with a lack of metabolic plasticity in maternal monocytes at T3. Extracellular acidification rate (ECAR) measurements were reduced with pregravid obesity both at baseline (10% lower; Figure 6A) as well as following LPS stimulation and glucose injection (45% increase in leans vs. 15% in obese; Figure 6B). Furthermore, monocytes from subjects with obesity accumulated 10% higher levels of neutral lipids (Figure 6C). These data indicate that pregravid obesity is associated with a reduced preference for aerobic glycolysis in monocytes at T3 and suggest metabolic constraints on immune activation in line with a refractory/immune-tolerant phenotype.

Additionally, we detected changes in effector functions in monocytes with pregravid obesity. Fewer cells from the obese group phagocytosed *E.coli* (Figure 6D, left), albeit the number of pathogen particles per cell was significantly higher (7%) (Figure 6D, right) in the obese group. Furthermore, pregravid obesity resulted in a 25% increase in cytosolic ROS following LPS stimulation (Figures 6F and S8A). We conclude that maternal obesity reduces the functional plasticity of circulating monocytes, impacting their ability to produce cytokines and engulf bacteria.

### Maternal obesity alters the differentiation trajectory of circulating monocytes

Monocytes under a state of endotoxin tolerance (reduced responsiveness to LPS challenge) or recovering from tolerance demonstrate defects in differentiation and polarization trajectory (O'Carroll et al., 2014). Therefore, we next investigated the impact of pregravid obesity on macrophage polarization potential using *in vitro* differentiation of monocytes collected at T3 using IFNγ (M1 like) and IL-4 (M2 like) conditioning (Figure 6F). CD64 expression was significantly reduced on monocyte-derived macrophages from mothers with obesity both after 7 days of differentiation (10-fold lower) as well as after IFNγ stimulation (2-fold lower) (Figure 6G, left). Macrophages from mothers with obesity failed to induce CD86 upregulation following IFNγ stimulation (Figure 6G, right), while HLA-DR induction was comparable between the two groups (Figure S8B).

In line with attenuated M1 skewing, secretion of pro-inflammatory cytokines TNFα and IL-12p70 (2-fold reduction) (Figure 6H) and chemokines CXCL9 (2-fold decrease) (Figure S8C) and CXCL11 (3-fold reduction) (Figure S8D) was reduced following IFNγ stimulation. Although we observed no differences in the expression of M2-like macrophage surface markers CD163 (Figure S8E) and CD209 (Figure S8F) following IL-4 conditioning, we observed significantly lower levels (50% reduction) of secreted regulatory molecules IL-10 (Figure 6E), CCL11 (Figure S8G) and PDGF-BB (Figure S8H).

## Discussion

Maternal obesity poses significant risks to maternal health, including increased susceptibility to infections during pregnancy (Robinson et al., 2005; Sebire et al., 2001; Stapleton et al., 2005), labor (Acosta et al., 2012; McLean et al., 2012; Salim et al., 2012), and post-partum (Acosta et al., 2012; McLean et al., 2012; Salim et al., 2012), suggesting disruptions in the pregnancy “immune clock”. This phenomenon refers to precisely timed adaptations in the maternal immune system, notably increased neutrophil numbers, enhanced innate immune responses, augmented STAT signaling in CD4+ T cells and NK cells, and significant changes in the circulating proteome (Aghaeepour et al., 2017, 2018; Le Gars et al., 2016). Previous studies profiling the impact of pregravid obesity on maternal peripheral immune responses have focused on one particular type of measurement at one stage of pregnancy (Acosta et al., 2012; McLean et al., 2012; Sureshchandra et al., 2018). Therefore, in this study, we used a systems approach to characterize the impact of pregravid obesity on pregnancy-associated “immunological clock” using a combination of proteomic, functional, and genomic assays.

Profiling longitudinal differences in plasma inflammatory factors between the first (T1) and third (T3) trimesters in lean subjects and those with pregravid obesity (BMI >30) showed that independent of maternal BMI, the transition from T1 to T3 was associated with increased levels of several pro-inflammatory (TNFα, S100B, IL-2, IFNβ, IL-17A, IL-18, IL-23) and regulatory (IL-4, IL-10, and IL-13) factors. These observations are consistent with previous reports of systemic immune activation and counter-regulation over the course of pregnancy (Aghaeepour et al., 2017). In addition to these analytes, and as previously reported (Basu et al., 2011a; Catalano et al., 2009; Challier et al., 2008; Friis et al., 2013; Roberts et al., 2011; Sen et al., 2014; Stewart et al., 2007), pregravid obesity resulted in significantly higher levels of IL-6 and CRP both at T1 and T3, highlighting a state of chronic low-grade inflammation. Higher levels of IL-6 and CRP have been linked to obstetric complications (Christian and Porter, 2014; Stewart et al., 2007).

Plasma levels of insulin, leptin, and adiponectin also increased with gestational age in lean subjects, likely a consequence of metabolic adaptations to the nutritional demands of a growing fetus. In contrast, in subjects with obesity, systemic levels of insulin, leptin, and resistin did not increase with gestational age, in line with reduced weight gain in this group (Table 1 and (Lindsay et al., 2018)). Aberrant insulin and leptin levels have been associated with both miscarriage (Baban et al., 2010) and preeclampsia (Laivuori et al., 2000), both of which are increased with pregravid obesity.

Profiling circulating immune cell subsets at T1 and T3 revealed modest cellular adaptations. Pregnancy was associated with increased total WBC counts, as previously reported (Chandra et al., 2012). However, at both T1 and T3, mothers with obesity had higher WBC counts relative to lean mothers. This significant increase was contributed by higher granulocyte counts, as previously described for non-gravid obese individuals (Rosales, 2018). No differences in T or B cell frequencies were observed with obesity, but a higher frequency of IL-4 producing CD4 T cells was noted with pregnancy regardless of maternal BMI indicative of a Th2 bias with pregnancy (Marzi et al., 1996). The most significant change with pregnancy was an increase in CD56^++^CD16^+^ NK cells. This subset of regulatory NK cells has been shown to increase with gestational age, and is the dominant NK cell subset (>95%) in the placental decidua (Le Bouteiller, 2013). As reported in previous studies (Aghaeepour et al., 2017), no significant differences were seen in other innate immune cell subsets.

Interestingly, levels of plasma regulatory factors (IL1RA, PDGF, CCL22) were significantly elevated, whereas those of IL-27, which activates monocytes via the STAT1 signaling pathway, were lower in plasma of mothers with obesity at T3. Moreover, circulating pro-inflammatory IL-1β increased with gestation in lean mothers but remained unchanged in mothers with obesity. Additionally, levels of CCL2 and CRP decreased with gestational age in mothers with obesity. Collectively, these observations strongly suggest that pregravid obesity disrupts changes in immune factors associated with myeloid cell activation.

Indeed, pregravid obesity was associated with a lower frequency of nonclassical (CD16+) monocytes and lower expression of HLA-DR on monocytes at T3. Comparison of monocyte transcriptomes at T3 from lean mothers and those with obesity using high-resolution single-cell RNA-sequencing (scRNA-Seq) confirmed a significant reduction in the number of nonclassical monocytes, and expression of both *CD16* and *HLA-DR*. Additionally, scRNA-Seq analysis showed increased expression of genes encoding inflammatory molecules *LYZ*, *VCAN*, and *HLA-DR* in monocytes from lean subjects, while expression of antiviral genes was suppressed in monocytes from obese subjects. Additionally, regulatory genes such as *CD163*, *AHR*, and *CD52* were increased with pregravid obesity.

These transcriptional changes were mirrored by functional differences in monocytes obtained from lean subjects and those with obesity at T3. Specifically, in lean subjects, activation of circulating monocytes increased between T1 and T3 as indicated by increased frequency of IL-6/TNFα producing cells following LPS stimulation and elevated levels of plasma sCD14. Moreover, bulk RNA-Seq revealed an enhanced transcriptional response to LPS at T3 relative to T1 in the lean subjects relative to those with obesity marked by higher expression of pro-inflammatory molecules (*IL6*, *IL1B*, *TNF*) and induction of transcription factors (RELA, IRF1, STAT3). These observations are in line with earlier studies that have demonstrated that monocyte responses to influenza are enhanced with gestational age (Le Gars et al., 2016). Enhancement of innate immune responses throughout pregnancy might be a mechanism to counter dampened T cell responses and maintain anti-microbial immunity in the pregnant woman.

Pregravid obesity disrupted pregnancy-associated monocyte activation, leading to a significantly reduced number of TNFα/IL-6 producing monocytes relative to lean subjects in response to LPS at T3. Similarly, RNA-Seq analysis revealed dampened induction of cytokine and chemokine genes and a failure to up-regulate expression of key transcription factors NFKB1 and FOSB following LPS stimulation. The dampened monocyte response is further evident from reduced surface expression of activation markers CD40 and CD86. Collectively, these differences in the innate immune system provide a potential explanation for the increased incidence of infections in obese pregnant women. It is possible that the state of chronic low-grade inflammation associated with obesity in combination with a pregnancy-induced increase in circulating immune mediators results in a state of endotoxin tolerance in circulating monocytes. The lack of maternal monocyte activation at T3 with pregravid obesity may indicate an adaptation aimed at protecting the developing fetus from maternal obesity-induced chronic low-grade inflammation.

Several factors have been proposed to be responsible for monocyte activation with pregnancy, including elevated levels of leptin and other cytokines (Sacks et al., 2001), exposure to placental microparticles released into circulation by syncytial trophoblasts (Redman et al., 2012), fetal DNA (Bianchi et al., 1996), or circulation of peripheral monocytes through the placenta (Mellembakken et al., 2002). Our RNA-Seq analysis also indicated changes in the expression of various epigenetic modifying factors. In line with these transcriptional changes, we report a significant increase in chromatin accessibility within the promoter and intergenic loci that regulate inflammatory responses between T1 and T3 in resting monocytes from lean subjects. Epigenetic changes with healthy pregnancy have been shown in human uterine NK cells (Gamliel et al., 2018) and mouse mammary glands (Dos Santos et al., 2015). Our study is the first to highlight chromatin remodeling in circulating immune cells, providing the first clues of an epigenetic clock of human pregnancy. While the precise mechanisms regulating this epigenetic clock are not clear, studies have demonstrated that both type I interferons and TNF can cooperatively reprogram the epigenome of myeloid cells resulting in increased chromatin accessibility at inflammatory loci (Park et al., 2017) and the establishment of transcriptional memory at the chromatin level. Interestingly, our analysis of circulating immune mediators shows a dramatic increase in plasma levels of both TNF and type-I interferons with pregnancy.

Pregravid obesity disrupted this epigenetic clock, resulting in a failure to induce transcription of genes important for chromatin remodeling following LPS stimulation at T3. Indeed, minimal chromatin changes were noted in monocytes from subjects with obesity between T1 and T3. A direct comparison of profiles at T1 and T3 shows a lack of remodeling within promoter and enhancer regions that regulate cytokine-signaling (*TNF*), myeloid cell activation (*CD80*), and immune effector process (*HLA-DRA*) in monocytes from subjects with obesity. Moreover, regions accessible in monocytes from lean subjects (but remained closed in obese subjects) contained putative binding sites for transcription factors that orchestrate pro-inflammatory responses (AP-1, IRF1, IRF3, and IRF8). In contrast, regions accessible in monocytes from subjects with obesity harbored binding sites for regulatory factors such as STAT6 and SMAD2. These epigenetic changes closely correlated with functional changes observed at T3. For example, significant closing of *FCGR3A* locus in the obese group at T3 was associated with a drop in frequencies of nonclassical monocytes, reduced surface expression of CD16 on total monocytes, and reduced uptake of *E. coli*. More importantly, our analysis of epigenetic changes (increased closing of loci associated with myeloid cell activation and cytokine production) in subjects with obesity at T3 correlate swith dampened cytokine responses to LPS stimulation and M1/M2 differentiation *ex vivo*. Finally, the increased opening of sites over-representative of M2-like macrophage identity correlates with defective M1-like polarization observed in *in vitro* differentiation assay. These observations suggest epigenetic constraints induced by pregravid obesity.

Innate immune tolerance is a functional adaptive program within innate immune cells that limits transcriptional activation following restimulation. Epigenetic priming mechanisms play critical roles in innate immune tolerance and training of innate immune memory (Divangahi et al., 2021), and our findings support the hypothesis that pregravid obesity results in LPS tolerance in circulating monocytes. Specifically, expression of *HLA-DR* in monocytes at T3 measured at the protein, transcript, and epigenetic levels were significantly reduced. Lower MHC class II molecule expression has been described in models of endotoxin tolerance and sepsis patients during the late stages of immunoparalysis (Wolk et al., 2003). Interestingly, monocytes from pregnant subjects with obesity had an enhanced oxidative burst following LPS exposure. This observation contrasts studies describing reduced *ex vivo E. coli* and *Staphylococcus aureus* induced ROS production in tolerant monocytes (Grondman et al., 2019). These differences in outcomes could be explained by different triggers of oxidative stress (LPS vs. pathogen). Innate immune tolerance to LPS can also impact the ability of monocytes to differentiate toward M1-or M2-like states under polarizing conditions (Divangahi et al., 2021). In agreement with transcriptional and cytokine responses, monocyte-derived macrophages (MoDM) from mothers with obesity polarized poorly to M1 but not to M2 skewing conditions.

Another manifestation of endotoxin tolerance in monocytes is the lack of metabolic plasticity. Indeed, monocytes obtained from humans injected with low dose LPS show a lack of metabolic plasticity, failing to up-regulate glycolysis, pentose phosphate pathway (PPP), and down-regulating lipid metabolic pathways when re-challenged *ex vivo* with LPS (Grondman et al., 2019). Our analysis of maternal monocytes at T3 showed reduced basal extracellular acidification rate (ECAR) with obesity indicative of dampened preference for glycolysis. Additionally, we observed poor induction of glycolysis following LPS injection. This coupled with the accumulation of higher levels of neutral lipids further supported metabolic reprogramming with maternal obesity.

**Figure 7:**
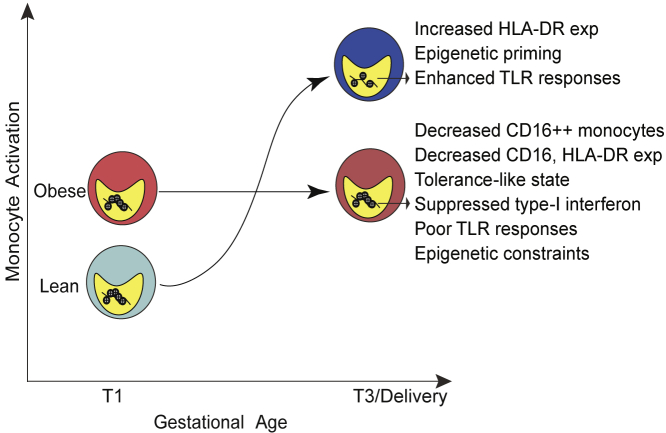
Model of the trajectory of monocyte activation with gestation and its disruption by pregravid obesity

In summary, this study revealed that pregravid obesity disrupts the monocyte activation trajectory associated with pregnancy (Figure 7), thereby providing a potential explanation for increased susceptibility to infections during gestation and post-partum in pregnant subjects with obesity. The study also provides a conceptual framework of an epigenetic clock that supports the immune clock of pregnancy. We demonstrated that pregravid obesity disrupts the immune clock of pregnancy, altering the metabolic, molecular, and functional phenotype of peripheral monocytes toward a regulatory phenotype. Future studies will focus on generating a comprehensive model of monocyte activation with additional gestational time points and assessing the impact of pregravid obesity on the monocyte response to pathogens. Peripheral blood monocytes are recruited to replenish the macrophage pool in placental decidua; hence, future experiments will also interrogate the impact of pregravid obesity at the maternal fetal interface.

### Limitations of the study

Lack of blood sampling during the second and early third trimesters precluded us from modeling temporal changes in immune responses over the entire course of pregnancy. Additionally, baseline readouts would have facilitated a better understanding of immune dysregulation due to obesity versus a combination of pregnancy and obesity. Another major limitation in the study is the relatively small number of samples collected at both T1 and T3, which would have allowed a more robust longitudinal analysis.

## STAR★Methods

### Key resources table

**Table:** 

REAGENT or RESOURCE	SOURCE	IDENTIFIER
**Antibodies**		

CD3-PE	BD Pharmigen	Cat#556612 Clone-SP34
CD19-PE	Biolegend	Cat#302208 Clone-HIB19
CD16-PB	Biolegend	Cat#302032 Clone-3G8
CD11c-PE-Cy7	Biolegend	Cat#301608 Clone-3.9
CD14-AF700	Biolegend	Cat#301822 Clone-M5E2
CD123-PCP-Cy5.5	Biolegend	Cat#306016 Clone-6H6
CD56-BV711	Biolegend	Cat#318336 Clone-HCD56
HLA-DR-APC-Cy7	Biolegend	Cat#307618 Clone-L243
CCR5-PB	Biolegend	Cat#359124 Clone-J418F1
TLR2-AF488	Biolegend	Cat#309712 Clone-TLR2.1
TLR4-BV711	BD Biosciences	Cat#564404 Clone-TF901
CD163-PCP-Cy5.5	Biolegend	Cat#333608 Clone-GHI/61
CCR2-BV605	Biolegend	Cat#357124 Clone-K036C2
CD11c-PE-eF610	Invitrogen	Cat#61-0116-42 Clone-3.9
CD11b-PE-Cy7	Biolegend	Cat#301322 Clone-ICRF44
CX3CR1-PE	Biolegend	Cat#341604 Clone-2A9-1
CD14-APC-Cy7	Biolegend	Cat#301820 Clone-M5E2
HLA-DR-PCP-Cy5.5	eBioscience	Cat#45-9956-73 Clone-LN3
TNF-APC	Invitrogen	Cat#17-7349-82 Clone-MAB11
IL-6-PE	BD Biosciences	Cat#551473 Clone-MQ2-6A3
CD62L-FITC	Biolegend	Cat#304805 Clone-DREG-56
CD40-BV510	Biolegend	Cat#334330 Clone-5C3
CD80-PE-Cy7	Biolegend	Cat#305218 Clone-2D10
CD83-PCP-Cy5.5	Biolegend	Cat#305320 Clone-HB15e
CD86-BV605	Biolegend	Cat#305430 Clone-IT2.2
CD64-BV711	Biolegend	Cat#305042 Clone-10.1
CXCR6-PE	Biolegend	Cat #356004 Clone-K041E5
PD-L1-PE-Dazzle594	Biolegend	Cat#329732
Human CD14 Microbeads	Miltenyi Biotec	Cat#130-118-906
CD14-FITC	Biolegend	Cat#301304 Clone-M5E2
CD209-PE	Biolegend	Cat#343004 Clone-DCS-8C1
Dynabeads Human T-Activator CD3/CD28	Gibco	Cat#111.32D

**Biological samples**		

TLR4 Agonist - Ultrapure LPS from E. coli 055:B5	Invivogen	cat#tlrl-pb5lps
Human AB Serum	Omega Scientific	Cat#HS-20
Fetal Bovine Serum, USDA Certified, Heat Inactivated	Omega Scientific	Cat#FB-02
FetalPlex™Animal Serum Complex	GeminiBio	Cat#100-602

**Chemicals, peptides, and recombinant proteins**		

Recombinant Human IFNγ	Peprotech	Cat#300-02 Source: *E. coli*
Recombinant Human IL-4	Peprotech	Cat#200-04 Source: *E. coli*
SYTOX Blue Dead Cell Stain	ThermoFisher Scientific	Cat#S34857
BODIPY™ 493/503 (4,4-Difluoro-1,3,5,7,8-Pentamethyl-4-Bora-3a,4a-Diaza-*s*-Indacene)	ThermoFisher	Cat#D3922
AMPure XP Beads	Beckman Coulter	Cat#A63881
TRIS, 1.0M buffer soln., pH 6.8	Alfa Aesar	Cat#J63831
Sodium chloride, Molecular Biology grade	Sigma-Aldrich	Cat#71376
Magnesium Chloride Hexahydrate	Fisher Scientific	Cat#BP214-500
Tagment DNA Enzyme 1	Illumina	Cat#15027865
TWEEN-20	Sigma-Aldrich	Cat#P9416-100ML
Digitonin	Promega	Cat#G9441
TD Tagment DNA Buffer	Illumina	Cat#15027866
Bovine Serum Albumin (BSA), Fraction V—Molecular Biology Grade	GeminiBio	Cat#700-106P

**Critical commercial assays**		

Customized Multiplexed Human Lumiex Kit (40-plex)	R & D Systems	Cytokines (IFNb, IFNg, IL-1b, IL-10, IL-12p70, IL-13, IL-15, IL-17A, IL-18, IL-1RA, IL-2, IL-21, IL-4, IL-5, IL-7, TNFa, IL-23, IL-31, IL-22, IL-27), chemokines (CCL2/MCP-1, CCL3/MIP-1a, CCL4/MIP-1b, CCL5/RANTES, CCL11/Eotaxin, CXCL1/GROa, CXCL8/IL-8, CXCL9/MIG CXCL10/IP-10, CXCL11/I-TAC, CXCL12/SDF-1a, CXCL13/BCA-1, growth factors (BDNF, GM-CSF, HGF, EGF, VEGF, PDGF-BB) and additional molecules (PD-L1, S100).
Millipore Human 3-plex Metabolic Panel	Millipore Sigma	Cat#HMHEMAG-34K
Millipore Human 5-plex Adipokine Panel	Millipore Sigma	Cat#HADK1MAG-61K
Human sCD14 ELISA kit	Hycult Biotech	Cat#HK320-02
Human CRP ELISA kit	Invitrogen	Cat#88-7502-28
Human IL-6 High Sensitivity ELISA kit	Invitrogen	Cat#BMS213HS
RNeasy Micro Kit for RNA extractions	QIAGEN	Cat#74004
TruSeq®Stranded mRNA Library Prep	Illumina	Cat#20020595
Chromium Single Cell 3ʹReagent Kits v3	10X Genomics	Cat#PN-1000075
Agilent Seahorse XFp Glycolytic Rate Assay Kit	Agilent Technologies	Cat#103346-100
Agilent Seahorse XFp Glycolytic Stress Assay Kit	Agilent	Cat#103017-100
CellROX™Deep Red Reagent	ThermoFisher Scientific	Cat#C10422
pHrodo™ Red *E. coli* BioParticles™Conjugate for Phagocytosis	ThermoFisher Scientific	Cat#P35361
Human Premixed 29-plex Magnetic Luminex Assay	R&D Systems	Cat#Custom-LxSA-H-29 Lot#C0003514
Agilent High Sensitivity DNA Kit	Agilent Technologies	Cat#5067-4626
Agilent RNA 6000 Nano Kit	Agilent Technologies	Cat#5067-1511

**Deposited data**		

Bulk RNA and ATAC-Seq data	This paper	NCBI Sequence Read Archive: PRJNA722536
Single cell RNA-Seq data	This paper	NCBI Sequence Read Archive: PRJNA725222
Flow Cytometry data	This paper	Flowrepository.org Repository IDs FR-FCM-Z3RM, FR-FCM-Z3RP, FR-FCM-Z3RS

**Software and algorithms**		

Prism	GraphPad	Version#8
FASTQC	Babraham Bioinformatics	Version#0.11.9
Trim Galore	Babraham Bioinformatics	Version#0.6.5
GenomicRanges	R (Bioconductor)	Version#1.40.0
edgeR	R (Bioconductor)	Version#3.30.3
DESeq2	R (Bioconductor)	Version#1.28.1
CellRanger	10X Genomics	Version#4.0.0
Seurat	R package	Version#3.1.5
Metascape	www.metascape.org	
Seahorse Wave	Agilent Technologies	Version#2.6.1
Bowtie2	http://bowtie-bio.sourceforge.net/	Version#2.4.2
Picard	https://broadinstitute.github.io/picard/	Version#2.21.3
Samtools	http://www.htslib.org/	Version#1.9
ATACseqQC	R (Bioconductor)	Version#1.14.4
HOMER	homer.ucsd.edu	Version#4.11
DAVID	https://david.ncifcrf.gov/	Version#6.8
ggplot2	R package	Version#3.3.3
Gplots	R package	Version#3.1.1
GenomicFeatures	R (Bioconductor)	Version#1.40.1
systemPipeR	R (Bioconductor)	Version#1.22.0
Attune NxT Flow Cytometer Software	ThermoFisher Scientific	Version#2.5
xPONENT® Software for Luminex Instruments	Luminex Corp	Version#4.3

### Resource availability

#### Lead contact

Further information and request for resources and reagents should be directed to and will be fulfilled by the Lead Contact, Dr. Ilhem Messaoudi (imessaou@uci.edu).

#### Materials availability

The study did not generate new unique reagents.

#### Data and code availability

The datasets supporting the conclusions of this article are available on NCBI’s Sequence Read Archive – PRJNA722536 (RNA-Seq and ATAC-Seq), PRJNA725222 (single-cell RNA-Seq). Flow cytometry data has been made available on FlowRepository (flowrepository.org, repository IDs FR-FCM-Z3RM, FR-FCM-Z3RP, FR-FCM-Z3RS). All additional data and code are available from authors upon request.

### Human subjects

This study was approved by the Institutional Ethics Review Board of Oregon Health and Science University and the University of California, Irvine. Written consent was obtained from all subjects. A total of 117 non-smoking women (69 lean and 48 obese) who had an uncomplicated, singleton pregnancy were enrolled for this study. The breakdown is as follows: 1) 37 women classified as lean and 28 women classified as obese were enrolled during the first trimester (∼week 12, designated as T1) of whom 29 lean and 27 obese also provided a blood sample during the third trimester (∼week 37, designated as T3); 2) 32 women classified as lean and 20 women classified as obese based on pre-pregnancy BMI were enrolled during the third trimester and provided one sample at ∼37 weeks (T3). The mean age of the 69 lean women was 32.8±4.7 years, and the average pre-pregnancy BMI was 21.9 ± 1.7 kg/m^2^; the mean age of the 48 obese women was 29.4±9 years with an average pre-pregnancy BMI of 35.2 ± 5.2 kg/m^2^ (Table 1). The racial distribution of the entire cohort was as follows: 93 Caucasian, 7 Asian American, 2 Pacific Islander, 6 American Indian/Alaskan native, 6 African American, and 3 declined to report. Only healthy pregnant women with no known diagnosis of pre-gestational diabetes or medications for chronic conditions aside from thyroid, anti-depressant, or anti-coagulation were included in this study. Additional exclusion criteria included active maternal infection, documented fetal congenital anomalies, substance abuse, preeclampsia, gestational diabetes, gestational hypertension, chorioamnionitis, significant medical conditions (active cancers, cardiac, renal, hepatic, or pulmonary diseases), pre-term deliveries, and an abnormal glucose tolerance test. Women underwent a fasting blood draw and body composition via air displacement plethysmography using a BodPod (Life Measurement Inc). These 117 subjects were a subset from a larger cohort that we recently described in greater detail (Damen et al., 2021; Marshall et al., 2019, 2020). No significant differences in race/ethnicity breakdown, parity, gestational age at delivery, mode of delivery, fetal birth weight, or fetal sex were found between the lean subjects and those with obesity (Table 1).

### Method details

#### Plasma and peripheral blood mononuclear cell (PBMC) isolation

Complete blood counts were obtained by Beckman Coulter hematology analyzer (Brea, CA). Peripheral blood mononuclear cells (PBMC) and plasma were obtained by standard density gradient centrifugation over Ficoll (GE Healthcare). PBMC were frozen in 10% DMSO/FBS and stored in liquid nitrogen until analysis. Plasma was stored at −80°C until analysis.

#### Luminex assay

Immune mediators in plasma were measured using a customized multiplex human factor panel (R & D Systems, Minneapolis MN) measuring cytokines (IFNβ, IFNγ, IL-1β, IL-10, IL-12p70, IL-13, IL-15, IL-17A, IL-18, IL-1RA, IL-2, IL-21, IL-4, IL-5, IL-7, TNFα, IL-23, IL-31, IL-22, IL-27), chemokines (CCL2/MCP-1, CCL3/MIP-1α, CCL4/MIP-1β, CCL5/RANTES, CCL11/Eotaxin, CXCL1/GROα, CXCL8/IL-8, CXCL9/MIG CXCL10/IP-10, CXCL11/I-TAC, CXCL12/SDF-1α, CXCL13/BCA-1, growth factors (BDNF, GM-CSF, HGF, EGF, VEGF, PDGF-BB) and additional molecules (PD-L1, S100). Metabolic hormones were measured using a 3-plex kit measuring insulin, leptin, and PYY (Millipore, Burlington, MA). Adipokines were assayed using a 5-plex kit measuring adiponectin, adipsin, lipocalin-2, total PAI-1, and resistin (Millipore, Burlington, MA). Samples were diluted per the manufacturer’s instructions and run in duplicates on the Magpix Instrument (Luminex, Austin, TX). Data were fit using a 5P-logistic regression on xPONENT software. Group differences were compared using one-way ANOVA for unpaired samples followed by Holm-Sidak multiple test correction.

#### ELISA

CRP and IL-6 were measured using a high-sensitivity ELISA (Life Technologies, Carlsbad, CA). Plasma soluble CD14 (sCD14) was measured using ELISA per manufacturer’s instruction (Hycult Biotech, Uden, Netherlands). Group differences were compared using one-way ANOVA for unpaired samples followed by Holm-Sidak multiple test correction.

#### PBMC and monocyte phenotyping

10^6^ PBMC were stained using the following cocktail of antibodies to enumerate innate immune cells and their subsets: PE-CD3, PE-CD19, PB-CD16, PE-Cy7-CD11c, AF700-CD14, PCP-Cy5.5-CD123, BV711-CD56, and APC-Cy7-HLA-DR. An additional 10^6^ PBMC from a subset of samples were stained using an additional panel to characterize monocyte phenotype: AF700-CD14, APC-Cy7-HLA-DR, PB-CCR5, AF488-TLR2, BV711-TLR4, PCP-Cy5.5-CD163, BV605-CCR2, PE-eF610-CD11c, PE-Cy7-CD11b, and PE-CX3CR1. After surface staining, cell pellets were washed twice in PBS and resuspended in cold FACS buffer (PBS with 2% FBS and 1mM EDTA). All samples were acquired with the Attune NxT Flow Cytometer (ThermoFisher Scientific, Waltham, MA) and analyzed using FlowJo 10.5.0 (Ashland, OR). Group differences were compared using one-way ANOVA for unpaired samples followed by Holm-Sidak multiple test correction.

#### Intracellular cytokine assay and monocyte activation

Roughly 10^6^ PBMC were stimulated for 16hr at 37°C in RPMI supplemented with 10% FBS in the presence or absence of 1 ug/mL LPS (TLR4 ligand, *E.coli* 055:B5; Invivogen, San Diego CA); Brefeldin A (Sigma, St. Louis MO) was added after 1 hour incubation. Cells were stained for APC-Cy7-CD14 and PCP-Cy5.5-HLA-DR, fixed, permeabilized, and stained intracellularly for APC-TNFα and PE-IL-6. A subset of PBMC samples were stimulated with LPS for 16hr without Brefeldin A, washed twice with PBS and surface stained using a cocktail containing the following antibodies for 20 minutes: FITC-CD62L, AF700-CD14, HLA-DR-APC-Cy7, PB-CD16, BV510-CD40, PE-Cy7-CD80, PCP-Cy5.5-CD83, BV605-CD86, BV711-CD64, PE-CXCR6, and PE-Dazzle594-PD-L1. Cell pellets were washed twice in PBS, and resuspended in cold FACS buffer (PBS with 2% FBS and 1mM EDTA). All samples were acquired with the Attune NxT Flow Cytometer (ThermoFisher Scientific, Waltham, MA) and analyzed using FlowJo 10.5 (Ashland, OR). Group differences were compared using one-way ANOVA for unpaired samples followed by Holm-Sidak multiple test correction.

#### Isolation of monocytes

Monocytes were purified from freshly thawed PBMC using CD14 antibodies conjugated to magnetic microbeads per the manufacturer’s recommendations (Miltenyi Biotec, San Diego, CA). Magnetically bound monocytes were washed and eluted for collection. Purity was assessed using flow cytometry and was on average ≥95% (Figure S7A).

#### Monocyte stimulation

10^5^ purified monocytes were cultured in RPMI supplemented with 10% FBS with or without 1 ug/mL LPS (TLR4 ligand, Ultrapure *E. coli* 055:B5; Invivogen, San Diego CA) in 96-well tissue culture plates at 37C in a 5% CO_2_ environment for 8 hr. Plates were spun down, and cell pellets were resuspended in Qiazol (Qiagen) for RNA extraction. Both cells and supernatants were stored at −80C until they could be processed as a batch.

#### Library generation for bulk RNA-Seq

Total RNA was isolated from monocytes using mRNeasy kit (Qiagen, Valencia, CA). Quality and concentrations were measured using Agilent 2100 Bioanalyzer. Libraries were generated using TruSeq Stranded Total RNA-Seq kit (Illumina, San Diego, CA). Briefly, following rRNA depletion, mRNA was fragmented for 8 min, converted to double-stranded cDNA, and adapter ligated. Fragments were then enriched by PCR amplification and purified. Size and quality of the library were verified using Qubit and Bioanalyzer. Libraries were multiplexed and sequenced on the HiSeq4000 platform (Illumina, San Diego CA) to yield an average of 20 million 100 bp single-end reads per sample.

#### Bulk RNA-seq analysis

Quality control of raw reads was performed using FASTQC and TrimGalore, retaining bases with quality scores of ≥20 and length ≥35 base pair. Reads were aligned to human genome (hg38) using splice aware aligner TopHat using annotations available from ENSEMBL (GRCh38.85) database. Quantification of read counts was performed using GenomicRanges package in R and normalized to derive transcripts per million (TPM) counts. Lowly expressed genes were filtered at the counting stage, eliminating ones with 0 counts in at least 3 samples regardless of the group. To detect the effect of pregravid obesity on the transcriptome of resting monocytes at T1 and T3 respectively, differential expression analysis was performed using quasi-likelihood linear modeling in edgeR. To test pairwise longitudinal changes within lean and obese groups, data were fit into negative binomial GLMs with a design matrix that preserved sample ID information. Due to the relatively low number of genes passing FDR cutoff <0.05, differentially expressed genes (DEGs) with a statistical p-value <0.01 were included in subsequent analyses.

Responses to LPS were modeled pairwise at each time point using negative binomial GLMs following low read count filtering. DEGs were defined as genes with expression difference FDR <0.05. Functional enrichment of DEG was performed using Metascape and InnateDB. Transcription factors that regulate the expression of DEG were predicted using ChEA3 tool using ENSEMBL ChIP database. Heatmaps of fold change or TPMs and bubble plots of enrichment of Transcription Factor (TF) regulation or Gene Ontology (GO) terms were generated using ggplot in R. Gene expression data have been deposited in NCBI’s Sequence Read Archive (SRA accession number PRJNA722536).

#### Cell Sorting and library generation for single-cell (sc)RNA-seq

PBMC from delivery time point were thawed, and live cells were sorted into RPMI (supplemented with 30% FBS) using the BD FACS Aria Fusion and SYTOX Blue (1:1000, ThermoFisher). Sorted cells were counted in triplicates and resuspended in PBS with 0.04% BSA in a final concentration of 1200 cells/uL. Cells were immediately loaded in the 10X Genomics Chromium with a loading target of 17,600 cells. Libraries were generated using the V3 chemistry per the manufacturer’s instructions (10X Genomics, Pleasanton CA). Libraries were sequenced on Illumina NovaSeq with a sequencing target of 50,000 reads per cell.

#### scRNA-seq data analysis

Raw reads were aligned and quantified using the Cell Ranger Single-Cell Software Suite (version 3.0.1, 10X Genomics) against the GRCh38 human reference genome using the STAR aligner. Downstream processing of aligned reads was performed using Seurat (version 3.1.1). Droplets with ambient RNA (<400 detected genes), potential doublets (cells with more than 4000 detected genes), and dying cells (>20% total mitochondrial gene expression) were excluded during initial QC. Data objects from the lean and obese groups were integrated using Seurat. Data normalization and variance stabilization were performed using *SCTransform* function using a regularized negative binomial regression, correcting for differential effects of mitochondrial and ribosomal gene expression levels and cell cycle. Dimension reduction was performed using the *RunPCA* function to obtain the first 30 principal components followed by clustering using the *FindClusters* function in Seurat. Visualization of clusters was performed using UMAP algorithm as implemented by Seurat’s *runUMAP* function. Cell types were assigned to individual clusters using *FindMarkers* function with a fold change cutoff of at least 0.5 and using a known catalog of well-characterized scRNA markers for PBMC (Zheng et al., 2017).

Three monocyte clusters expressing high levels of *CD14* were extracted from dimension-reduced Seurat objects using the *subset* function. Cells were re-clustered and visualized using UMAP. Doublet clusters were identified by iterative clustering removing clusters with high expression of genes associated with T cells, B cells, and NK cells. Differential expression analysis was tested using the Wilcoxon rank sum test followed by Bonferroni correction using all genes in the dataset. For gene scoring analysis, we compared gene signatures and pathways from KEGG (https://www.genome.jp/kegg/pathway.html) in subpopulations using Seurat’s *AddModuleScore* function. Two-way functional enrichment of differential signatures was performed on Metascape. Differential hierarchies within the monocyte compartment were reconstructed using Monocle (version 2.8.0). Briefly, clustering was performed using t-SNE, and differential genes identified using Monocle’s *differentialGeneTest*. These genes (q-value < 1e-10) were used for ordering cells on a pseudotime. Raw fastq files have been deposited in NCBI’s Sequence Read Archive (SRA accession number PRJNA725222).

#### Neutral lipid staining

Neutral lipids in monocytes were quantified in monocytes using flow cytometry. Briefly, 500,000 PBMC were surface-stained (CD14-AF700, HLA-DR-APC-Cy7) for 20 minutes at 4°C, washed twice, and resuspended in 500 uL warm 1X PBS containing 1 ug/mL BODIPY™ 493/503 (ThermoFisher Scientific). Cells were incubated at 37°C for 10 minutes, and samples were acquired with the Attune NxT Flow Cytometer (ThermoFisher Scientific, Waltham MA) and analyzed using FlowJo 10.5 (Ashland OR). Group differences were compared using an unpaired t-test with Welch’s correction.

#### Metabolic assays

Basal Oxygen Consumption Rate (OCR) and Extracellular Acidification Rate (ECAR) were measured using Seahorse XF Glycolysis Rate Assay on Seahorse XFp Flux Analyzer (Agilent Technologies) following manufacturer’s instructions. Briefly, 200,000 purified monocytes were seeded on Cell-Tak (Corning) coated 8-well culture plates in phenol free RPMI media containing 2 mM L-glutamine, 10 mM L-glucose, 1mM sodium pyruvate, and 5mM HEPES. Seeded plates were placed in 37C incubator without CO_2_ and run on the XFp with extended basal measurements, followed by serial injections of Rotenone/Actinomycin (final well concentration 0.5 uM) to block oxidative phosphorylation followed by 2-Deoxy-D-Glucose (2-DG, 500 mM) to block glycolysis. Cellular responses to stress under activated conditions were measured using Seahorse XF Glycolysis Stress Assay. Purified monocytes were seeded in the in glucose free media and cultured in the presence/absence of 1 ug/mL LPS for 1 hr in 37°C incubator without CO_2_. Plates were run on the XFp for 8 cycles of basal measurements, followed by acute injection of L-glucose (100 mM), oligomycin (50 uM), and 2-DG (500 mM). Data were analyzed on Seahorse Wave desktop software (Agilent Technologies). Aggregate differences were compared using an unpaired t-test with Welch’s correction.

#### Phagocytosis assay

Cellular phagocytosis was measured using pH-sensitive pHrodo®E. coli BioParticles® conjugates (ThermoFisher Scientific). 500,000 PBMC were activated with 100 ng/mL LPS for 4hr, washed twice, and incubated for an additional 2 hr in media containing pHrodo conjugates at 1 mg/mL conjugates. Pellets were washed twice, surface stained (CD14-AF700, HLA-DR-APC-Cy7), and resuspended in ice-cold FACS buffer. All samples were acquired with the Attune NxT Flow Cytometer (ThermoFisher Scientific, Waltham MA) and analyzed using FlowJo 10.5 (Ashland OR). Group differences were compared using an unpaired t-test with Welch’s correction.

#### Cellular ROS assay

For intracellular ROS measurements, 500,000 PBMC were stimulated with 1 ug/mL LPS for 4 hr. For negative control, cells were incubated in serum-free media containing 200 uM anti-oxidant N-acetylcysteine (NAC) for 1.5 hr. Both negative and positive controls were incubated with tert-butyl hydroperoxide (TBHP) for 30 minutes to induce oxidative stress. All samples were then incubated with 2.5 uM CellROX Deep Red (Life Technology) at 37°C for 30 minutes, washed twice, surface stained (CD14-FITC, HLA-DR-PCP-Cy5.5), and samples were acquired with the Attune NxT Flow Cytometer (ThermoFisher Scientific, Waltham MA) and analyzed using FlowJo 10.5 (Ashland OR). Group differences were compared using an unpaired t-test with Welch’s correction.

#### *In vitro*macrophage differentiation

100,000 purified monocytes were cultured in 24-well tissue culture plates treated for increased attachment (VWR) in RPMI supplemented with 1% Human AB Serum (Omega Scientific) for 7 days with media supplemented on day 3. On day 7, cells were polarized to M1-like macrophages using 1 ug/mL LPS and 100 ng/mL IFNγ (Peprotech) or M2-like macrophages using 100 ng/mL IL-4 (Peprotech) and cultured for an additional 24 hours (day 8). Cell pellets from day 7 and day 8 were surface-stained (CD16-PB; CD64-BV711; HLA-DR-APC-Cy7; CD86-BV605; CD163-PCP-Cy5.5; CD209-R-PE) and analyzed using flow cytometry. Supernatants were collected and quantified for secreted cytokines and chemokines using a 29-plex Luminex assay (R & D Systems). Group differences were compared using one-way ANOVA for unpaired samples followed by Holm-Sidak multiple test correction.

#### Library generation for ATAC-Seq

ATAC-Seq libraries were generated using a recently described modified protocol (OMNI-ATAC) to reduce mitochondrial reads. Briefly, 50,000 purified monocytes were lysed in lysis buffer (10mM Tris-HCl (pH 7.4), 10 mM NaCl, 3 mM MgCl_2_), for 3 min on ice to prepare the nuclei. Immediately after lysis, nuclei were spun at 500g for 10 min to remove the supernatant. Nuclei were then incubated with transposition mixture (100 nM Tn5 transposase, 0.1% Tween-20, 0.01% Digitonin and TD Buffer) at 37°C for 30 min. Transposed DNA was then purified with AMPure XP beads (Beckman Coulter) and partially amplified for 5 cycles using the following PCR conditions −72°C for 3 min; 98°C for 30s and thermocycling at 98°C for 10s, 63°C for 30s, and 72°C for 1 min. To avoid overamplification, qPCR was performed on 5 uL of partially amplified library. Additional cycles of amplification for the remainder of the sample were calculated from the saturation curves (cycles corresponding to a third of the saturation value). Fully amplified samples were purified with AMPure beads and quantified on the Bioanalyzer (Agilent Technologies, Santa Clara CA).

#### Analysis of ATAC-seq data

Paired reads from sequencing were quality checked using FASTQC and trimmed to retain reads with quality scores of ≥20 and minimum read lengths of 50 bp. Trimmed paired reads were aligned to the human genome (hg38) using Bowtie2 (–X 2000–k 1 –very-sensitive –no-discordant –no-mixed). Reads aligning to mitochondrial genome and allosomes were removed using samtools. PCR duplicate artifacts were then removed using Picard. Finally, poor quality alignments and improperly mapped reads were filtered using samtools (samtools view –q 10 –F 3844). To reflect the accurate read start site due to Tn5 insertion, BAM files were repositioned using ATACseqQC package in R. The positive and negative strands were offset by +4bp and -5bp, respectively. Samples within a group were merged and sorted using samtools.

Sample QC and statistics for merged BAM files were generated using HOMER makeTagDirectory function. Accessible chromatin peaks were called for mapped paired reads using HOMER findpeak function (-minDist 150 –region –fdr 0.05). PCA and sample clustering were performed on accessible peaks using DiffBind. Differentially accessible regions (DAR) in either direction were captured using HOMER getDiffererentialPeaks function (-q 0.05). DAR were annotated using the human GTF annotation file (GRCh38.85) and ChIPSeeker with a promoter definition of -1000 bp and +100 bp around the transcriptional start site (TSS). Peaks overlapping 5’UTRs, promoters, first exons, and first introns were pooled for functional enrichment of genes. For intergenic changes, the genes closest to the intergenic DAR were considered. Functional enrichment of this pooled list of genes was performed using DAVID (Fisher p-value <0.05). BAM files were converted to bigWig files using bedtools and visualized on the new WashU EpiGenome browser. ATAC seq data have been deposited in NCBI’s Sequence Read Archive (SRA accession number PRJNA722536).

### Quantification and statistical analysis

All statistical analyses were conducted in Prism 8 (GraphPad). All definitive outliers in two-way and four-way comparisons were identified using ROUT analysis (Q=0.1%). Data was then tested for normality using Shapiro-Wilk test (alpha=0.05). If data were normally distributed across all groups, differences with obesity and pregnancy were tested using ordinary one-way ANOVA with unmatched samples. Multiple comparisons were corrected using Holm-Sidak test, adjusting the family-wise significance and confidence level at 0.05. If gaussian assumption was not satisfied, differences were tested using Kruskall-Wallis test (alpha=0.05) followed by Dunn’s multiple hypothesis correction tests. Differences in normally distributed datasets were tested using an unpaired t-test with Welch’s correction (assuming different standard deviations). Two group comparisons that failed normality tests were carried out using the Mann-Whitney test. Associations and correlograms between BMI and cytokine levels were generated using corrplot package in R.

## Supplementalinformation

Document S1. Figures S1–S8

Table S1. Markers for identification of single cell clusters and markers differentially expressed with obesity at T3, related to Figure 3

Table S2. Differentially expressed genes in response to LPS, related to Figure 4

Table S3. Differentially accessible regions with pregnancy, related to Figure 5

Table S4. Differentially accessible regions with obesity at T1 and T3, related to Figure 5

## References

[bib1] Acosta C.D., Bhattacharya S., Tuffnell D., Kurinczuk J.J., Knight M. (2012). Maternal sepsis: a Scottish population-based case-control study. BJOG.

[bib2] Aghaeepour N., Ganio E.A., McIlwain D., Tsai A.S., Tingle M., Van Gassen S., Gaudilliere D.K., Baca Q., McNeil L., Okada R. (2017). An immune clock of human pregnancy. Sci. Immunol..

[bib3] Aghaeepour N., Lehallier B., Baca Q., Ganio E.A., Wong R.J., Ghaemi M.S., Culos A., El-Sayed Y.Y., Blumenfeld Y.J., Druzin M.L. (2018). A proteomic clock of human pregnancy. Am. J. Obstet. Gynecol..

[bib4] Aune D., Saugstad O.D., Henriksen T., Tonstad S. (2014). Maternal body mass index and the risk of fetal death, stillbirth, and infant death: a systematic review and meta-analysis. JAMA.

[bib5] Baban R.S., Ali N.M., Al-Moayed H.A. (2010). Serum leptin and insulin hormone level in recurrent pregnancy loss. Oman Med. J..

[bib6] Basu S., Haghiac M., Surace P., Challier J.C., Guerre-Millo M., Singh K., Waters T., Minium J., Presley L., Catalano P.M. (2011). Pregravid obesity associates with increased maternal endotoxemia and metabolic inflammation. Obesity (Silver Spring).

[bib7] Basu S., Leahy P., Challier J.C., Minium J., Catalano P., Hauguel-de Mouzon S. (2011). Molecular phenotype of monocytes at the maternal-fetal interface. Am. J. Obstet. Gynecol..

[bib8] Ben Amara A., Gorvel L., Baulan K., Derain-Court J., Buffat C., Verollet C., Textoris J., Ghigo E., Bretelle F., Maridonneau-Parini I. (2013). Placental macrophages are impaired in chorioamnionitis, an infectious pathology of the placenta. J. Immunol..

[bib9] Bianchi D.W., Zickwolf G.K., Weil G.J., Sylvester S., DeMaria M.A. (1996). Male fetal progenitor cells persist in maternal blood for as long as 27 years postpartum. Proc. Natl. Acad. Sci. U S A.

[bib10] Catalano P.M., Presley L., Minium J., Hauguel-de Mouzon S. (2009). Fetuses of obese mothers develop insulin resistance in utero. Diabetes Care.

[bib11] Challier J.C., Basu S., Bintein T., Minium J., Hotmire K., Catalano P.M., Hauguel-de Mouzon S. (2008). Obesity in pregnancy stimulates macrophage accumulation and inflammation in the placenta. Placenta.

[bib12] Chandra S., Tripathi A.K., Mishra S., Amzarul M., Vaish A.K. (2012). Physiological changes in hematological parameters during pregnancy. Indian J. Hematol.Blood Transfus..

[bib13] Christian L.M., Porter K. (2014). Longitudinal changes in serum proinflammatory markers across pregnancy and postpartum: effects of maternal body mass index. Cytokine.

[bib14] Chu S.Y., Callaghan W.M., Kim S.Y., Schmid C.H., Lau J., England L.J., Dietz P.M. (2007). Maternal obesity and risk of gestational diabetes mellitus. Diabetes Care.

[bib15] Cnattingius S., Villamor E., Johansson S., Edstedt Bonamy A.K., Persson M., Wikstrom A.K., Granath F. (2013). Maternal obesity and risk of preterm delivery. JAMA.

[bib16] Conner S.N., Verticchio J.C., Tuuli M.G., Odibo A.O., Macones G.A., Cahill A.G. (2014). Maternal obesity and risk of postcesarean wound complications. Am. J. Perinatol.

[bib17] Damen N.A., Gillingham M., Hansen J.G., Thornburg K.L., Purnell J.Q., Marshall N.E. (2021). Maternal dietary fat intake during pregnancy and newborn body composition. J. Perinatol.

[bib18] Divangahi M., Aaby P., Khader S.A., Barreiro L.B., Bekkering S., Chavakis T., van Crevel R., Curtis N., DiNardo A.R., Dominguez-Andres J. (2021). Trained immunity, tolerance, priming and differentiation: distinct immunological processes. Nat. Immunol..

[bib19] Dos Santos C.O., Dolzhenko E., Hodges E., Smith A.D., Hannon G.J. (2015). An epigenetic memory of pregnancy in the mouse mammary gland. Cell Rep.

[bib20] Faas M.M., de Vos P. (2017). Uterine NK cells and macrophages in pregnancy. Placenta.

[bib21] Faas M.M., Spaans F., De Vos P. (2014). Monocytes and macrophages in pregnancy and pre-eclampsia. Front Immunol..

[bib22] Flegal K.M., Kruszon-Moran D., Carroll M.D., Fryar C.D., Ogden C.L. (2016). Trends in obesity among adults in the United States, 2005 to 2014. JAMA.

[bib23] Friis C.M., Paasche Roland M.C., Godang K., Ueland T., Tanbo T., Bollerslev J., Henriksen T. (2013). Adiposity-related inflammation: effects of pregnancy. Obesity (Silver Spring).

[bib24] Gamliel M., Goldman-Wohl D., Isaacson B., Gur C., Stein N., Yamin R., Berger M., Grunewald M., Keshet E., Rais Y. (2018). Trained memory of human uterine NK cells enhances their function in subsequent pregnancies. Immunity.

[bib25] Gomez-Lopez N., StLouis D., Lehr M.A., Sanchez-Rodriguez E.N., Arenas-Hernandez M. (2014). Immune cells in term and preterm labor. Cell Mol Immunol.

[bib26] Grondman I., Arts R.J.W., Koch R.M., Leijte G.P., Gerretsen J., Bruse N., Kempkes R.W.M., Ter Horst R., Kox M., Pickkers P. (2019). Frontline Science: endotoxin-induced immunotolerance is associated with loss of monocyte metabolic plasticity and reduction of oxidative burst. J. Leukoc. Biol..

[bib27] Hadley E.E., Discacciati A., Costantine M.M., Munn M.B., Pacheco L.D., Saade G.R., Chiossi G. (2019). Maternal obesity is associated with chorioamnionitis and earlier indicated preterm delivery among expectantly managed women with preterm premature rupture of membranes. J. Matern.Fetal Neonatal. Med..

[bib28] Huda S.S., Forrest R., Paterson N., Jordan F., Sattar N., Freeman D.J. (2014). In preeclampsia, maternal third trimester subcutaneous adipocyte lipolysis is more resistant to suppression by insulin than in healthy pregnancy. Hypertension.

[bib29] Kay A.W., Fukuyama J., Aziz N., Dekker C.L., Mackey S., Swan G.E., Davis M.M., Holmes S., Blish C.A. (2014). Enhanced natural killer-cell and T-cell responses to influenza A virus during pregnancy. Proc. Natl. Acad. Sci. U S A..

[bib30] Kim S.S., Zhu Y., Grantz K.L., Hinkle S.N., Chen Z., Wallace M.E., Smarr M.M., Epps N.M., Mendola P. (2016). Obstetric and neonatal risks among obese women without chronic disease. Obstet. Gynecol..

[bib31] Laivuori H., Kaaja R., Koistinen H., Karonen S.L., Andersson S., Koivisto V., Ylikorkala O. (2000). Leptin during and after preeclamptic or normal pregnancy: its relation to serum insulin and insulin sensitivity. Metabolism.

[bib32] Le Bouteiller P. (2013). Human decidual NK cells: unique and tightly regulated effector functions in healthy and pathogen-infected pregnancies. Front Immunol..

[bib33] Le Gars M., Kay A.W., Bayless N.L., Aziz N., Dekker C.L., Swan G.E., Davis M.M., Blish C.A. (2016). Increased proinflammatory responses of monocytes and plasmacytoid dendritic cells to influenza Avirus infection during pregnancy. J.Infect Dis..

[bib34] Lindsay K.L., Brennan L., Rath A., Maguire O.C., Smith T., McAuliffe F.M. (2018). Gestational weight gain in obese pregnancy: impact on maternal and foetal metabolic parameters and birthweight. J. Obstet. Gynaecol..

[bib35] Luppi P., Haluszczak C., Betters D., Richard C.A., Trucco M., DeLoia J.A. (2002). Monocytes are progressively activated in the circulation of pregnant women. J. Leukoc. Biol..

[bib36] Luppi P., Haluszczak C., Trucco M., Deloia J.A. (2002). Normal pregnancy is associated with peripheral leukocyte activation. Am. J. Reprod.Immunol..

[bib37] Marshall N.E., Lallande L.F., Schedin P.J., Thornburg K.L., Purnell J.Q. (2020). Exclusive breastfeeding rates at 6 weeks postpartum as a function of preconception body mass index are not impacted by postpartum obstetrical practices or routines. Breastfeed. Med..

[bib38] Marshall N.E., Lau B., Purnell J.Q., Thornburg K.L. (2019). Impact of maternal obesity and breastfeeding intention on lactation intensity and duration. Matern.Child Nutr..

[bib39] Marzi M., Vigano A., Trabattoni D., Villa M.L., Salvaggio A., Clerici E., Clerici M. (1996). Characterization of type 1 and type 2 cytokine production profile in physiologic and pathologic human pregnancy. Clin. Exp. Immunol..

[bib40] McLean M., Hines R., Polinkovsky M., Stuebe A., Thorp J., Strauss R. (2012). Type of skin incision and wound complications in the obese parturient. Am. J. Perinatol.

[bib41] Mellembakken J.R., Aukrust P., Olafsen M.K., Ueland T., Hestdal K., Videm V. (2002). Activation of leukocytes during the uteroplacental passage in preeclampsia. Hypertension.

[bib42] Mor G., Abrahams V.M. (2003). Potential role of macrophages as immunoregulators of pregnancy. Reprod. Biol. Endocrinol..

[bib43] Mor G., Cardenas I. (2010). The immune system in pregnancy: a unique complexity. Am. J. Reprod.Immunol..

[bib44] Mor G., Cardenas I., Abrahams V., Guller S. (2011). Inflammation and pregnancy: the role of the immune system at the implantation site. Ann. N. Y Acad. Sci..

[bib45] Naccasha N., Gervasi M.T., Chaiworapongsa T., Berman S., Yoon B.H., Maymon E., Romero R. (2001). Phenotypic and metabolic characteristics of monocytes and granulocytes in normal pregnancy and maternal infection. Am. J. Obstet. Gynecol..

[bib46] O'Carroll C., Fagan A., Shanahan F., Carmody R.J. (2014). Identification of a unique hybrid macrophage-polarization state following recovery from lipopolysaccharide tolerance. J. Immunol..

[bib47] Park S.H., Kang K., Giannopoulou E., Qiao Y., Kang K., Kim G., Park-Min K.H., Ivashkiv L.B. (2017). Type I interferons and the cytokine TNF cooperatively reprogram the macrophage epigenome to promote inflammatory activation. Nat. Immunol..

[bib48] Polese B., Gridelet V., Araklioti E., Martens H., Perrier d'Hauterive S., Geenen V. (2014). The endocrine milieu and CD4 T-lymphocyte polarization during pregnancy. Front Endocrinol.(Lausanne).

[bib49] Redman C.W., Tannetta D.S., Dragovic R.A., Gardiner C., Southcombe J.H., Collett G.P., Sargent I.L. (2012). Review: does size matter? Placental debris and the pathophysiology of pre-eclampsia. Placenta.

[bib50] Roberts K.A., Riley S.C., Reynolds R.M., Barr S., Evans M., Statham A., Hor K., Jabbour H.N., Norman J.E., Denison F.C. (2011). Placental structure and inflammation in pregnancies associated with obesity. Placenta.

[bib51] Robinson H.E., O'Connell C.M., Joseph K.S., McLeod N.L. (2005). Maternal outcomes in pregnancies complicated by obesity. Obstet. Gynecol..

[bib52] Rosales C. (2018). Neutrophil: acell with many roles in inflammation or several cell types?. Front Physiol..

[bib53] Sacks G.P., Clover L.M., Bainbridge D.R., Redman C.W., Sargent I.L. (2001). Flow cytometric measurement of intracellular Th1 and Th2 cytokine production by human villous and extravillous cytotrophoblast. Placenta.

[bib54] Sacks G.P., Redman C.W., Sargent I.L. (2003). Monocytes are primed to produce the Th1 type cytokine IL-12 in normal human pregnancy: an intracellular flow cytometric analysis of peripheral blood mononuclear cells. Clin. Exp. Immunol..

[bib55] Sacks G.P., Studena K., Sargent K., Redman C.W. (1998). Normal pregnancy and preeclampsia both produce inflammatory changes in peripheral blood leukocytes akin to those of sepsis. Am. J. Obstet. Gynecol..

[bib56] Salim R., Braverman M., Teitler N., Berkovic I., Suliman A., Shalev E. (2012). Risk factors for infection following cesarean delivery: an interventional study. J. Matern.Fetal Neonatal.Med..

[bib57] Sebire N.J., Jolly M., Harris J.P., Wadsworth J., Joffe M., Beard R.W., Regan L., Robinson S. (2001). Maternal obesity and pregnancy outcome: a study of 287,213 pregnancies in London. Int. J. Obes.Relat.Metab.Disord..

[bib58] Sen S., Iyer C., Meydani S.N. (2014). Obesity during pregnancy alters maternal oxidant balance and micronutrient status. J. Perinatol.

[bib59] Stapleton R.D., Kahn J.M., Evans L.E., Critchlow C.W., Gardella C.M. (2005). Risk factors for group B streptococcal genitourinary tract colonization in pregnant women. Obstet. Gynecol..

[bib60] Stewart F.M., Freeman D.J., Ramsay J.E., Greer I.A., Caslake M., Ferrell W.R. (2007). Longitudinal assessment of maternal endothelial function and markers of inflammation and placental function throughout pregnancy in lean and obese mothers. J. Clin.Endocrinol.Metab..

[bib61] Sureshchandra S., Marshall N.E., Wilson R.M., Barr T., Rais M., Purnell J.Q., Thornburg K.L., Messaoudi I. (2018). Inflammatory determinants of pregravid obesity in placenta and peripheral blood. Front Physiol..

[bib62] Torloni M.R., Betran A.P., Horta B.L., Nakamura M.U., Atallah A.N., Moron A.F., Valente O. (2009). Prepregnancy BMI and the risk of gestational diabetes: a systematic review of the literature with meta-analysis. Obes. Rev..

[bib63] Wang Z., Wang P., Liu H., He X., Zhang J., Yan H., Xu D., Wang B. (2013). Maternal adiposity as an independent risk factor for pre-eclampsia: a meta-analysis of prospective cohort studies. Obes. Rev..

[bib64] Wolk K., Kunz S., Crompton N.E., Volk H.D., Sabat R. (2003). Multiple mechanisms of reduced major histocompatibility complex class II expression in endotoxin tolerance. J. Biol. Chem..

[bib65] Zheng G.X., Terry J.M., Belgrader P., Ryvkin P., Bent Z.W., Wilson R., Ziraldo S.B., Wheeler T.D., McDermott G.P., Zhu J. (2017). Massively parallel digital transcriptional profiling of single cells. Nat. Commun..

