# Supplementary material for: Functional and genomic adaptations of blood monocytes to pregravid obesity during pregnancy
Source: iScience. 2021 Jun 4;24(6):102690. doi: 10.1016/j.isci.2021.102690 (PMC8233196; doi:10.1016/j.isci.2021.102690)
Supplement: Document S1. Figures S1–S8 [file mmc1.pdf]

iScience, Volume *24*

Supplemental information

Functional and genomic adaptations of blood monocytes to pregravid obesity during pregnancy

Suhas Sureshchandra, Nicole E. Marshall, Norma Mendoza, Allen Jankeel, Michael Z. Zulu, and Ilhem Messaoudi

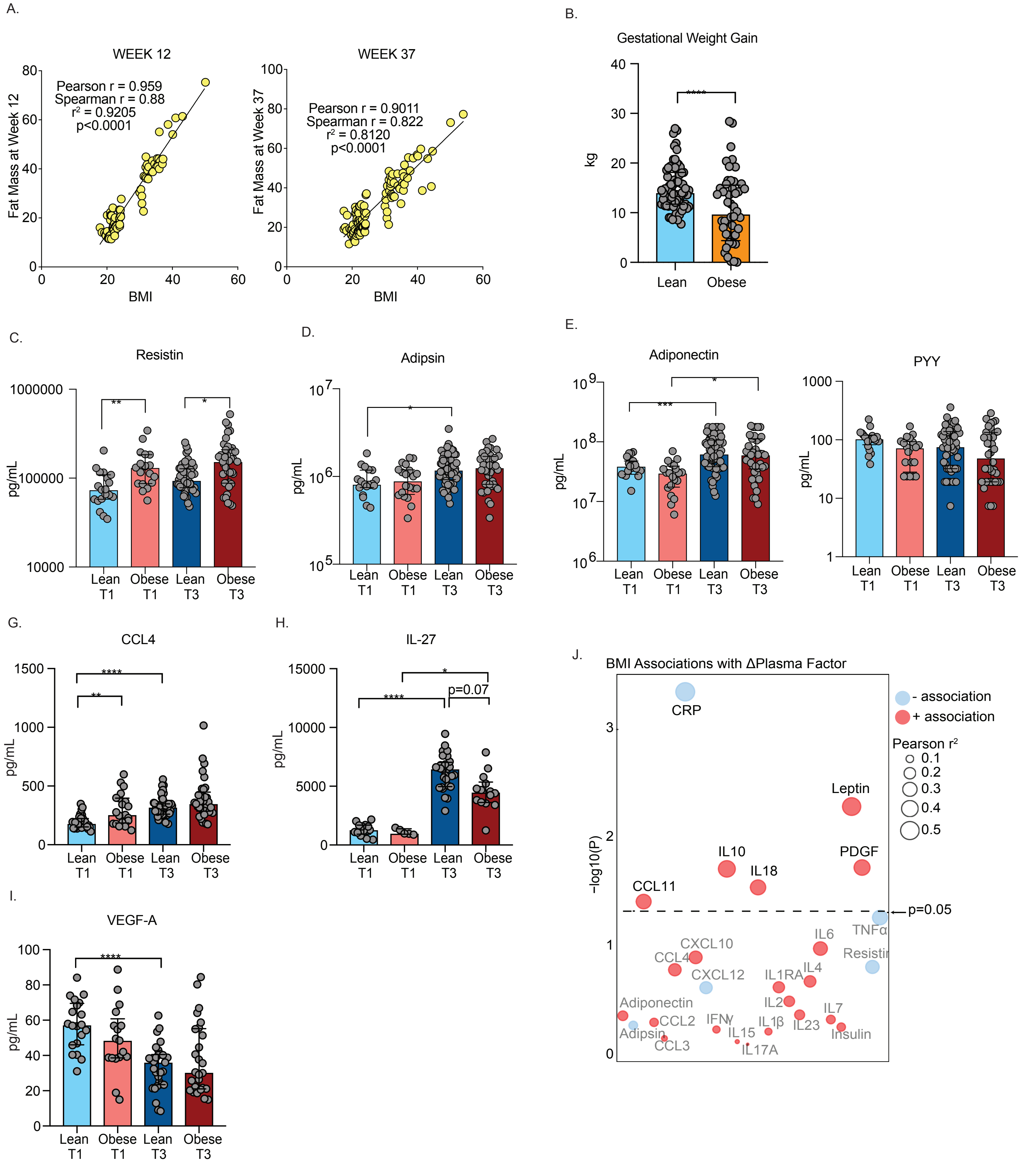

Figure S1: Longitudinal changes in circulating inflammatory environment (Related to Figure 1)

(A) Linear regression of fat mass and pregravid BMI at T1 and T3 (T1 n=71, T3 n=109). (B) Dot plots demonstrating gestational weight gain (GWG) in lean and obese subjects (Lean n=71, Obese n=48). (C-F) Bar graphs comparing circulating levels of (C) resistin, (D) adipsin, (E) adiponectin, and (F) peptide YY (PYY) at T1 and T3 (Lean T1 n=20; Obese T1 n=18, Lean T3 n=57, Obese T3 n=38). (G-I) Bar graphs comparing plasma levels of (G) IL-27, (H) CCL4, and (I) VEGF-A (Lean T1 n=20; Obese T1 n=17, Lean T3 n=29, Obese T3 n=26). (J) Association between changes in plasma analyte level with gestation (T3-T1) and BMI. The size of the bubble reflects the strength of Pearson's correlation, and color indicates the direction of association (red-positive; blue-negative).

Levels of significance: *- p<0.05, ** - p<0.01; *** - p<0.001; ****- p<0.0001.

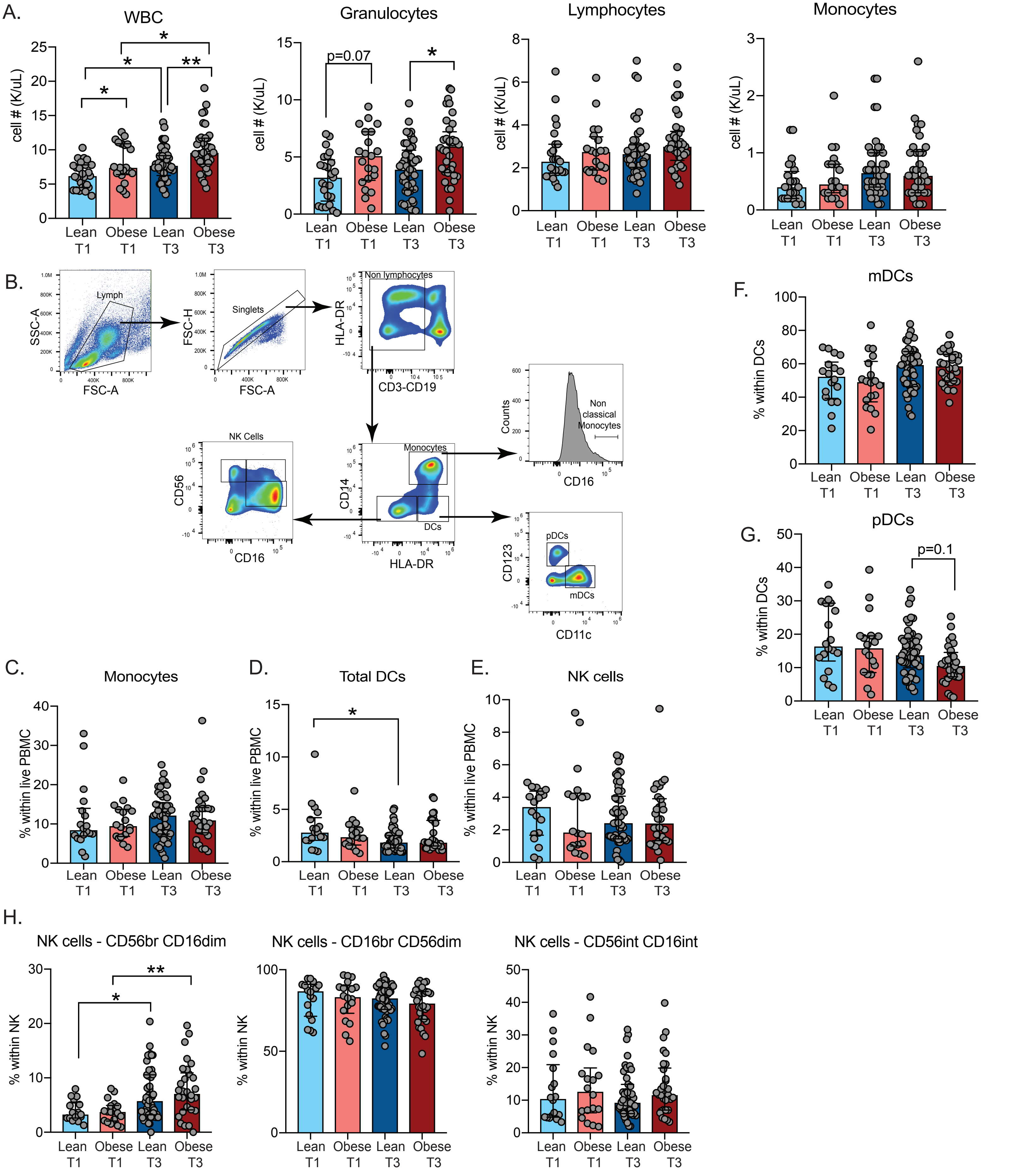

Figure S2: Immune cell profiling with pregnancy and obesity (Related to Figure 2)

(A) Complete blood counts of all blood samples analyzed (Lean T1 n=25; Obese T1 n=20, Lean T3 n=20, Obese T3 n=37) (B) Gating strategy for characterization of innate immune cell populations from PBMC. (C-E) Bar graphs comparing frequencies of total (C) monocytes, (D) dendritic cells, and (E) NK cells within PBMC. (F-G) Comparing frequencies of (F) myeloid DCs subsets, (G) plasmacytoid DCs, and (H) NK cell subsets (Lean T1 n=18; Obese T1 n=18, Lean T3 n=50, Obese T3 n=30).

Levels of significance: *- p<0.05, ** - p<0.01.

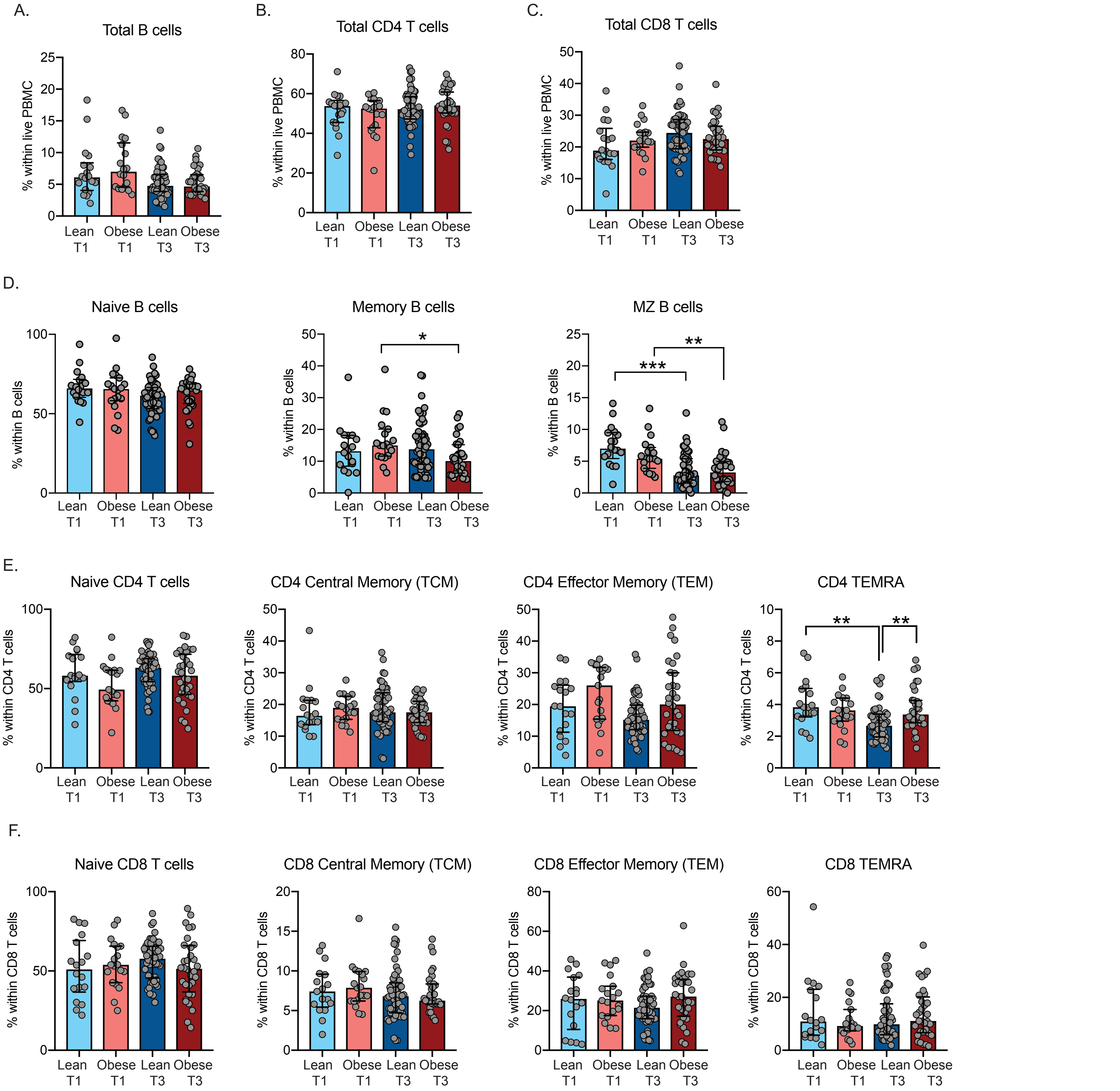

Figure S3: Phenotyping of adaptive immune cells (Related for Figure 2)
(A-C) Percentages of total (A) B cells, (B) CD4+ T cells, and (C) CD8+ T cells within PBMC. (D-F) Relative abundances of naïve and memory subpopulations with (D) B cells, (E) CD4+ T cells, and (F) CD8+ T cells (Lean T1 n=18; Obese T1 n=18, Lean T3 n=50, Obese T3 n=30).
Levels of significance: *- p<0.05, ** - p<0.01, **** - p<0.0001.

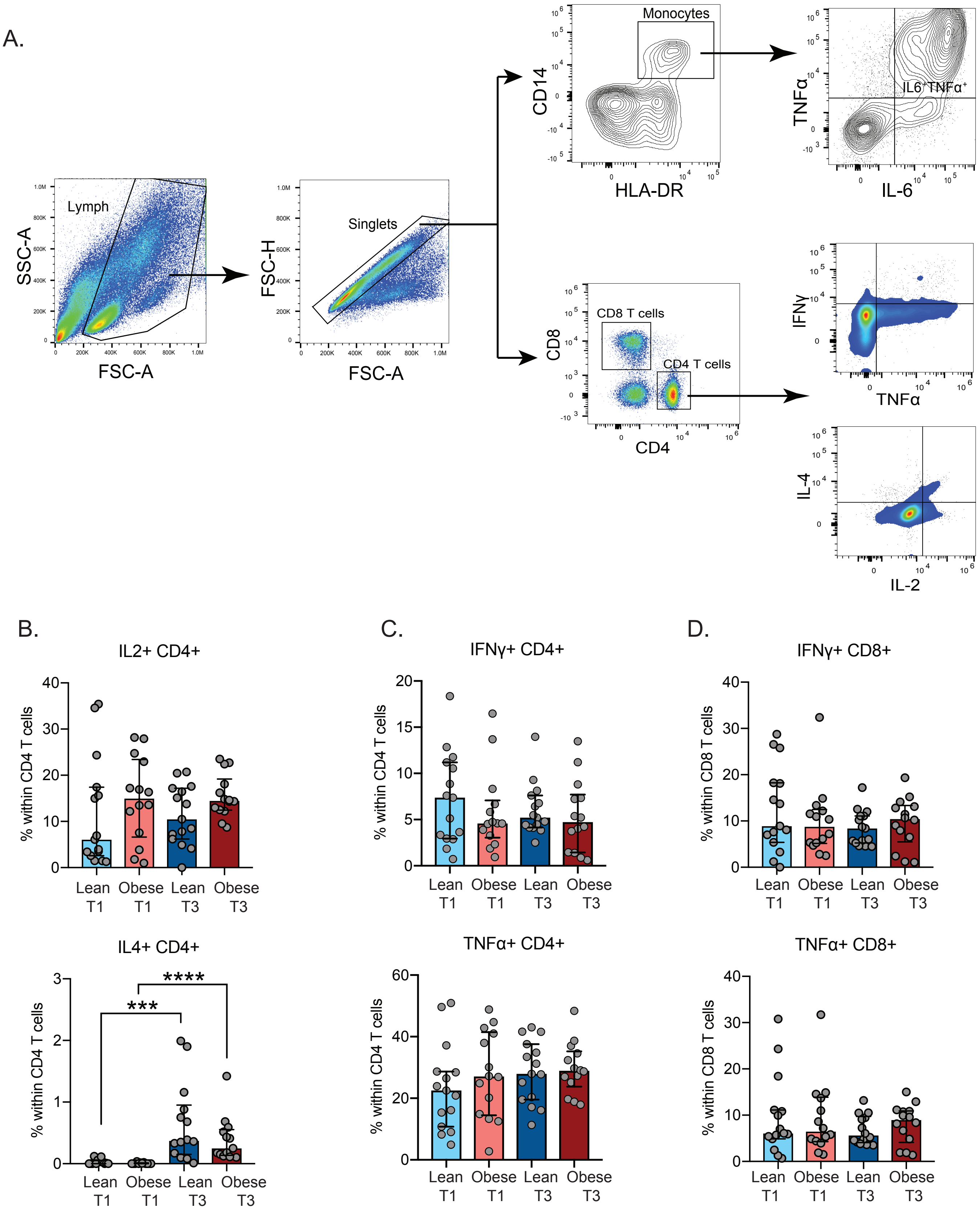

Figure S4: Cytokine responses to ex vivo stimulation (Related to Figure 2)

(A) Gating strategy for measuring frequencies of responding monocytes and T cells following ex vivo stimulation using intracellular cytokine staining. (B-D) Bar graphs responding T cells following CD3/CD28 stimulation. (B) Frequency of IL-2 (above) and IL-4 (below) producing CD4+ T cells. Frequency of IFNγ (above) and TNFα (below) producing (C) CD4+ and (D) CD8+ T cells (Lean T1/T3 n=15; Obese T1/T3 n=14).

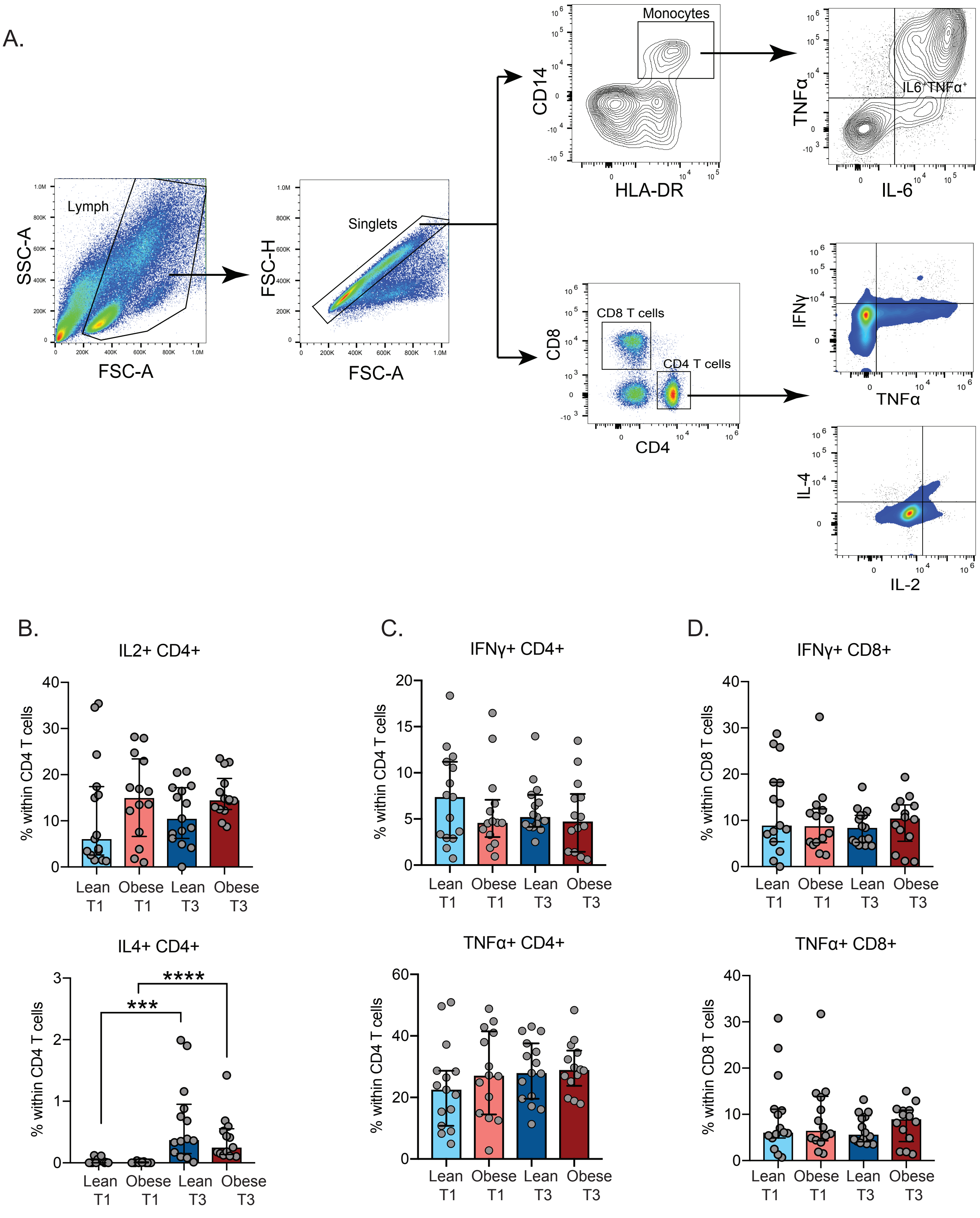

Figure S4: Cytokine responses to ex vivo stimulation (Related to Figure 2)

(A) Gating strategy for measuring frequencies of responding monocytes and T cells following ex vivo stimulation using intracellular cytokine staining. (B-D) Bar graphs responding T cells following CD3/CD28 stimulation. (B) Frequency of IL-2 (above) and IL-4 (below) producing CD4+ T cells. Frequency of IFNγ (above) and TNFα (below) producing (C) CD4+ and (D) CD8+ T cells (Lean T1/T3 n=15; Obese T1/T3 n=14).

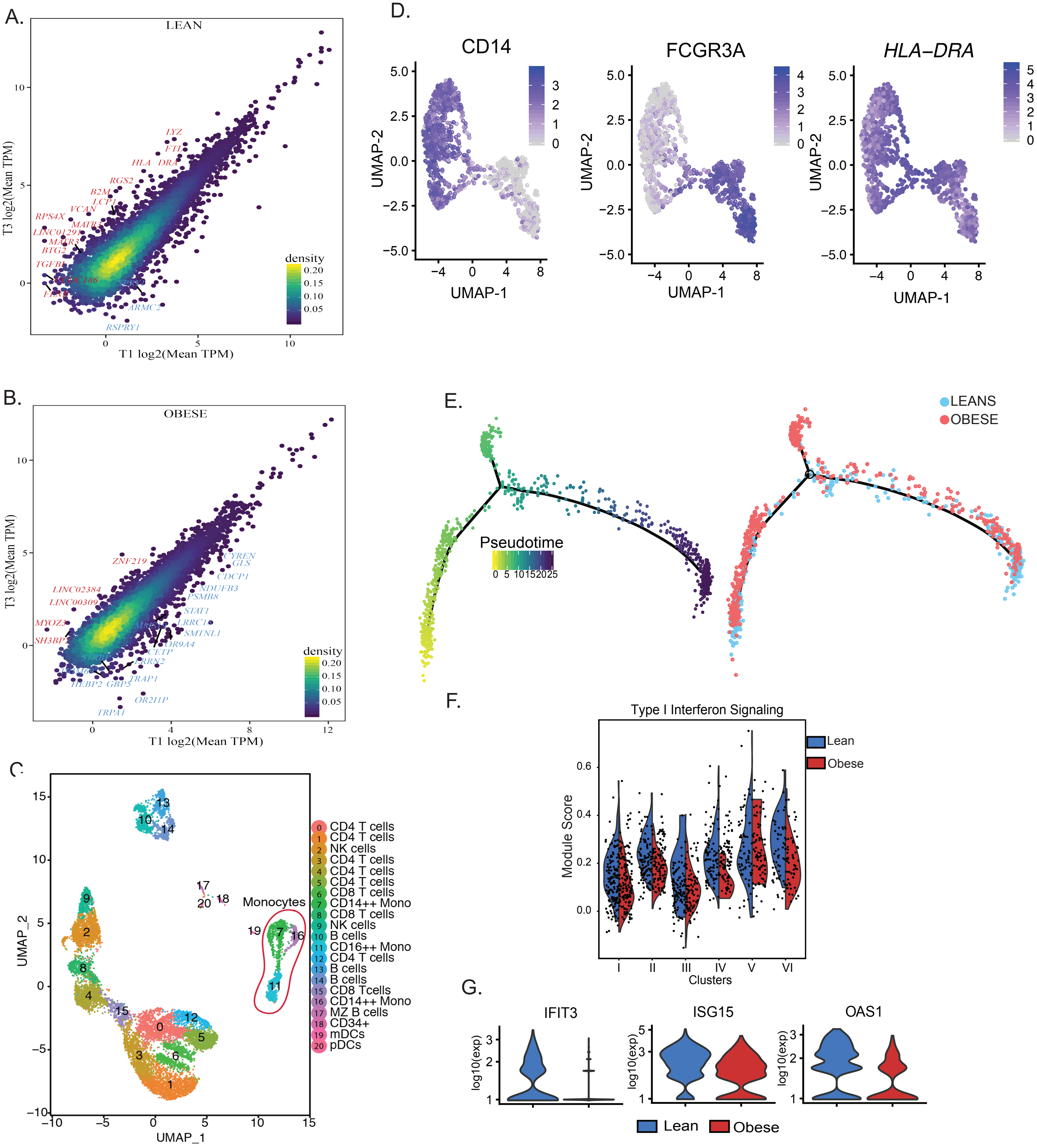

Figure S5: Baseline transcriptional differences with pregnancy (Related to Figure 3).

(A-B) Scatter plot depiction of normalized transcript counts (TPM) on a log2 scale from resting monocyte at T1 and T3 (projected on X and Y axes respectively) obtained from (A) lean (n=4/time point) and (B) obese (n=4/time point) groups. Only genes with significant expression differences (p<0.001) are annotated. (C) UMAP visualization of PBMC isolated from lean mothers (n=2) and mothers with obesity (n=2) at delivery time point. Live cells were enriched using FACS and profiled using droplet based single-cell RNA sequencing. (D) Feature plots of characteristic markers to delineate monocyte subsets based on their expression levels as a gradient of purple. (E) Pseudotime ordering of monocytes (left) revealing progressive shifts in cellular states with pregravid obesity (right). (F) Violin plots depicting score of type-I interferon signaling module across different clusters. (G) Normalized expression of select interferon-stimulated genes (ISGs) significantly down-regulated with maternal obesity.

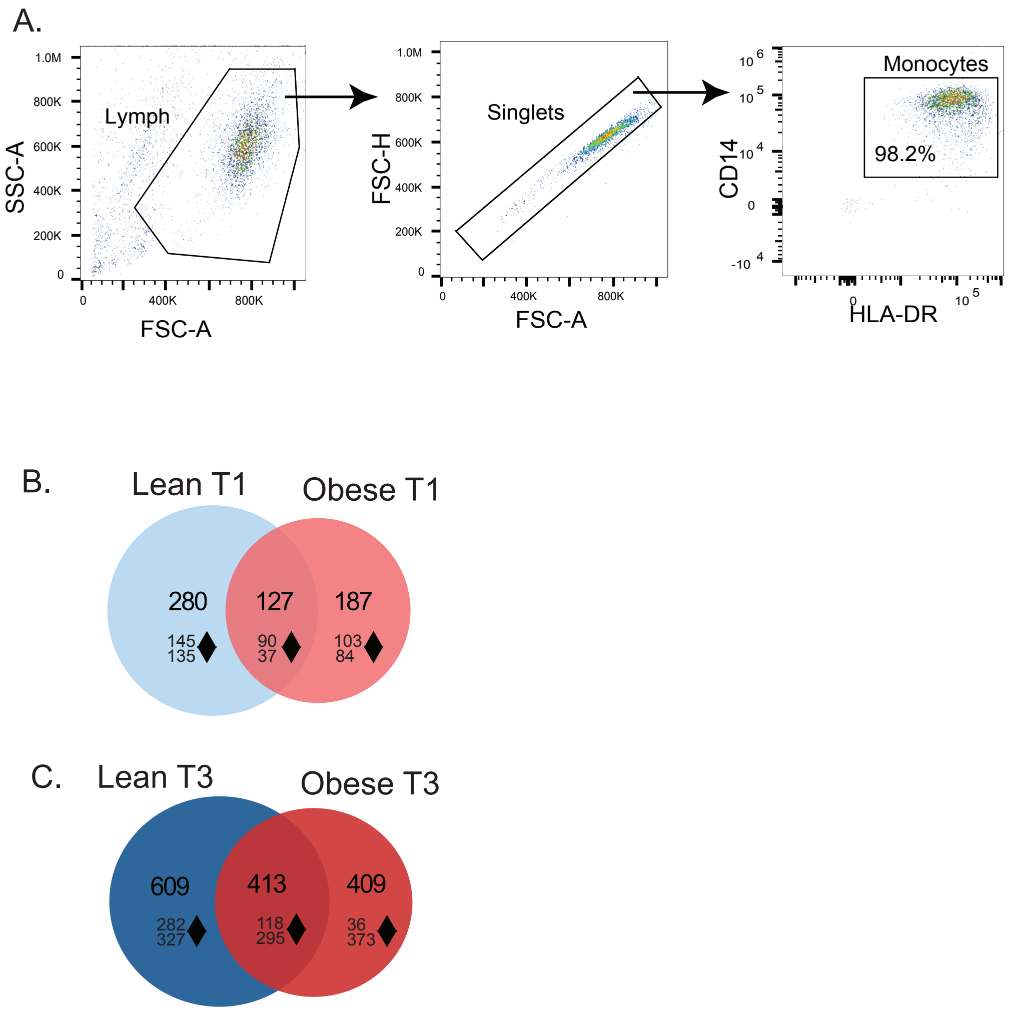

Figure S6: Profiling transcriptional responses to LPS (Related to Figure 4)
(A) Gating strategy for assessment of purity of monocytes following magnetic bead separation. (B) Comparing LPS responsive DEG in the lean and obese group at T1 (n=4/group) and (C) T3 (n=4/group).

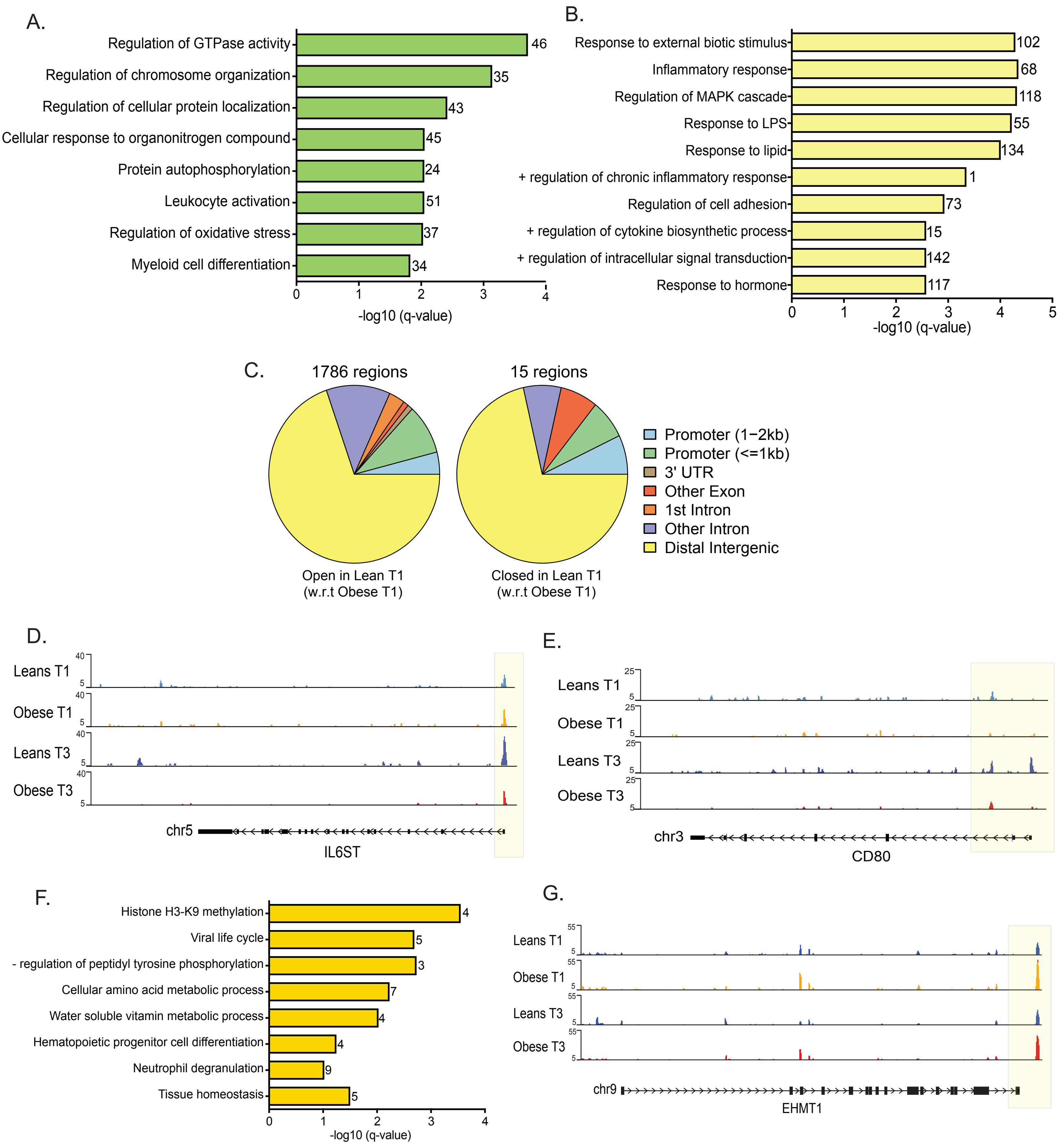

Figure S7: Epigenetic adaptations with pregnancy and obesity (Related to Figure 5)

(A) Functional enrichment of genes regulated by promoters and (B) intergenic regions spanning differentially accessible regions (DAR) open in the lean group at T3 relative to T1 (FC>2). The number of genes associated with these regions is indicated next to each GO term. (C) Genomic contexts of DAR when comparing monocyte ATAC peaks from lean and obese groups at T1. (D-E) WashU Epigenome tracks for (D) IL6ST and (E) CD80 locus with promoter vicinity highlighted in yellow. (F) Functional enrichment of genes regulated by intergenic DAR significantly open in the obese group relative to lean group at T3 identified by GREAT. (G) WashU Epigenome tracks for EHMT1 indicating greater accessibility in monocytes from subjects with obesity.

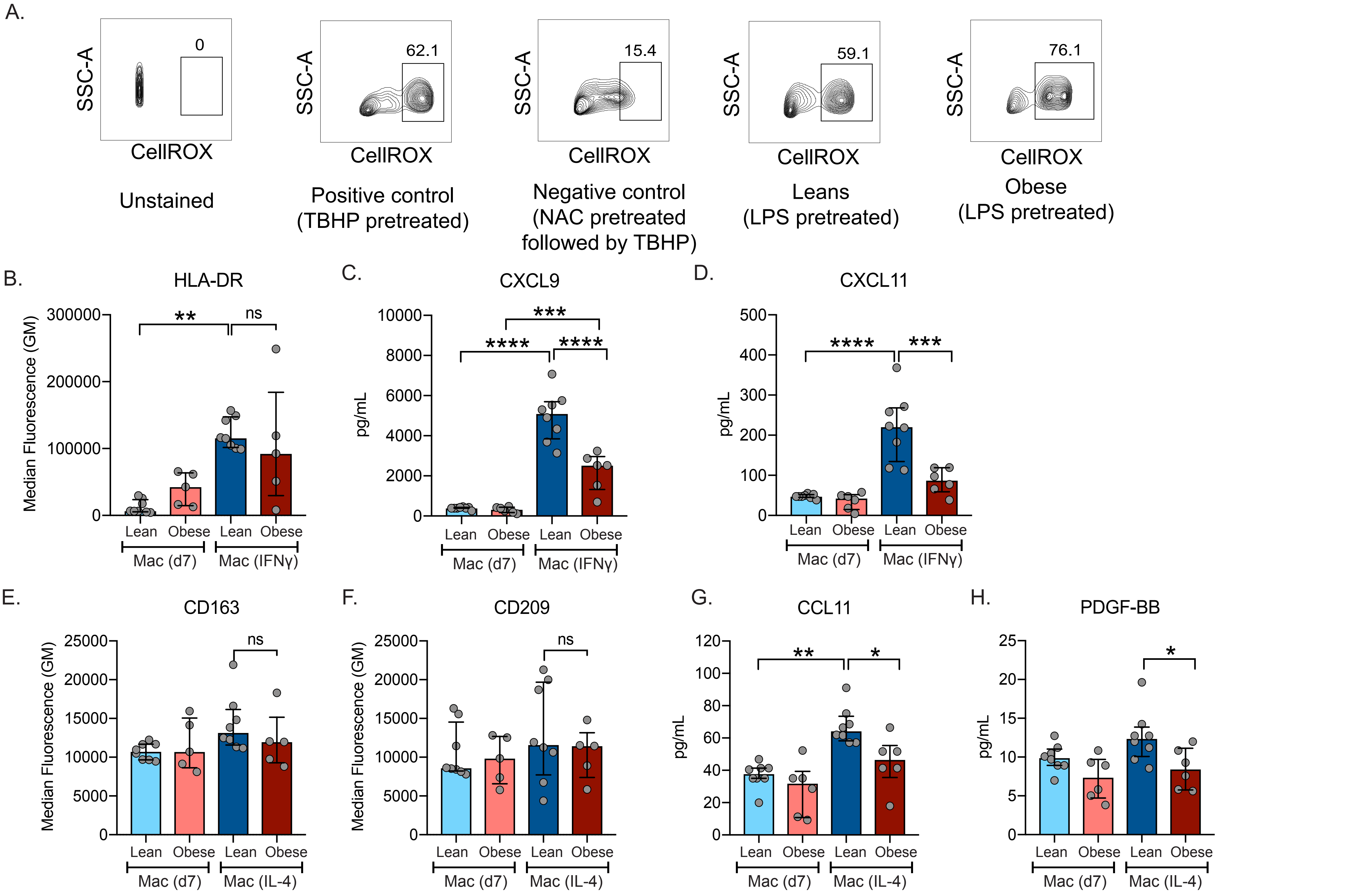

Figure S8: Functional rewiring of monocytes with maternal obesity at term (Related to Figure 6)

(A) Contour plots of CellROX signal from monocytes gated in lean and obese samples. NAC and TBHP treated monocytes serve as negative and positive controls, respectively. (B) Bar graphs comparing surface expression of HLA-DR and secreted levels of M1 associated chemokines (C) CXCL9 and (D) CXCL11 following LPS and IFNγ stimulation on day 7. (E) Bar graphs comparing surface expression of M2-associated markers CD163 and (F) CD209, and M2-associated chemokine (G) CCL11 (eotaxin) and growth factor (H) PDGF (leans n=8, obese n=5). Levels of significance: * - $p<0.05$; *** - $p<0.001$; ****-$p<0.0001$. Bars represent medians and interquartile ranges.
